# Machine learning-based identification of biomarkers and drugs in immunologically cold and hot pancreatic adenocarcinomas

**DOI:** 10.1186/s12967-024-05590-0

**Published:** 2024-08-16

**Authors:** Jia Ge, Juan Ge, Gu Tang, Dejun Xiong, Dongyan Zhu, Xiaoling Ding, Xiaorong Zhou, Mengmeng Sang

**Affiliations:** 1 Department of Immunology, School of Medicine, Nantong University, Nantong, 226001 China; 2 Department of Respiratory Medicine, Affiliated Nantong Hospital of Shanghai University, Nantong, 226011 China; 3 Department of Rehabilitation, the Second Affiliated Hospital of Nantong University, Nantong, 226001 China; 4 Department of Gastroenterology, Affiliated Hospital of Nantong University, Nantong, 226001 China

**Keywords:** Pancreatic adenocarcinoma, Hot/cold tumor, Machine learning, Prognostic model, Drug prediction, Molecular docking

## Abstract

**Background:**

Pancreatic adenocarcinomas (PAADs) often exhibit a “cold” or immunosuppressive tumor milieu, which is associated with resistance to immune checkpoint blockade therapy; however, the underlying mechanisms are incompletely understood. Here, we aimed to improve our understanding of the molecular mechanisms occurring in the tumor microenvironment and to identify biomarkers, therapeutic targets, and potential drugs to improve PAAD treatment.

**Methods:**

Patients were categorized according to immunologically hot or cold PAAD subtypes with distinct disease outcomes. Cox regression and weighted correlation network analysis were performed to construct a novel gene signature, referred to as ‘Downregulated in hot tumors, Prognostic, and Immune-Related Genes’ (DPIRGs), which was used to develop prognostic models for PAAD via machine learning (ML). The role of DPIRGs in PAAD was comprehensively analyzed, and biomarker genes able to distinguish PAAD immune subtypes and predict prognosis were identified by ML. The expression of biomarkers was verified using public single-cell transcriptomic and proteomic resources. Drug candidates for turning cold tumors hot and corresponding target proteins were identified via molecular docking studies.

**Results:**

Using the DPIRG signature as input data, a combination of survival random forest and partial least squares regression Cox was selected from 137 ML combinations to construct an optimized PAAD prognostic model. The effects and molecular mechanisms of DPIRGs were investigated by analysis of genetic/epigenetic alterations, immune infiltration, pathway enrichment, and miRNA regulation. Biomarkers and potential therapeutic targets, including *PLEC*, *TRPV1*, and *ITGB4*, among others, were identified, and the cell type-specific expression of the biomarkers was validated. Drug candidates, including thalidomide, SB-431542, and bleomycin A2, were identified based on their ability to modulate DPIRG expression favorably.

**Conclusions:**

By combining multiple ML algorithms, we developed a novel prognostic model with excellent performance in PAAD cohorts. ML also proved to be powerful for identifying biomarkers and potential targets for improved PAAD patient stratification and immunotherapy.

**Supplementary Information:**

The online version contains supplementary material available at 10.1186/s12967-024-05590-0.

## Introduction

Numerous studies have demonstrated that the immune composition of the tumor microenvironment (TME) plays a critical role in tumorigenesis and determines the response to immune checkpoint blockade (ICB) treatment [1–3]. CD8^+^ cytotoxic T lymphocytes (CTLs) and natural killer (NK) cells are major immune components that eliminate cancer cells, but their antitumor activity is constantly influenced, positively or negatively, by various cues from the TME. For example, dendritic cells, the most important antigen-presenting cells, are recruited into tumors by intratumoral NK cells and then activate CTLs by cross-presentation of neoantigens [4]. M1-type macrophages produce an array of proinflammatory cytokines, thereby indirectly promoting CTL and NK cell antitumor activity [5]. In contrast, some tumor stromal cells, such as M2-type tumor-associated macrophages, cancer-associated fibroblasts, and myeloid-derived suppressive cells, possess immunosuppressive properties; these cells inhibit the antitumor immune response, either by releasing immunosuppressive cytokines, such as IL-10 and transforming growth factor-beta (TGF-β), or by directly inhibiting CTL cytolytic activity through cell surface expression of coinhibitory molecules, such as PD-L1 and TIGIT [6, 7]. There is accumulating evidence that the TME is highly heterogeneous and significantly impacts the response of cancer patients to various treatments [8]; therefore, understanding the complex network underlying the immunosuppressive TME is crucial for patient stratification and individualized therapy.

Previous studies have classified tumors into immunologically “cold” and “hot” tumors, usually based on the level of immune infiltration in the TME [9]. Early-stage tumors generally contain more immune cells, particularly CD8^+^ T and NK cells, and increased proinflammatory cytokines and are more likely to respond to ICB treatment. In contrast, tumors that lack significant immune infiltration and are resistant to ICB treatment are usually referred to as cold tumors. Hence, distinguishing hot and cold tumors assists in the selection of patients for appropriate treatments; however, in practice, there is no clinically relevant consensus on the definition of hot/cold tumors and no biomarkers that can feasibly categorize patients with cancer into different immune subgroups, making it difficult to predict patient survival and response to ICB treatment in the clinic.

Machine learning (ML) algorithms allow computer systems to learn from input data to improve their performance. Furthermore, these algorithms enable computer systems to make clustering, optimization, and prediction decisions based on data without explicit programming. Several ML algorithms, including artificial neural network (ANN) [10], Boruta [11], random forest [12], support vector machine (SVM) [13], and extreme gradient boosting (XGBoost) [14], are widely employed to analyze gene expression data and biological characteristics. Genes crucial for predicting immunotherapy response or prognosis can be identified using ML algorithms, and by selecting the most informative genes, these algorithms decrease dimensionality and improve the efficiency of prognostic models [15, 16]. Moreover, ML algorithms can predict the genetic features and drug responses of patients, facilitating the development of personalized treatment plans [17].

Pancreatic adenocarcinoma (PAAD) is difficult to diagnose in its early stages and responds poorly to conventional treatment options, resulting in a 5-year survival rate of only 11% [18]. Despite the positive results of ICB therapy for some solid tumors, such as melanomas and non-small cell lung cancer, most patients with PAAD are inherently resistant to immunotherapy, but the mechanisms underlying this phenomenon are not fully understood [19]. In this study, we developed a novel immune-related gene signature and a consensus ML framework to search for the most favorable prognostic models for PAAD. Furthermore, we applied ML algorithms to identify biomarkers valuable for predicting survival and distinguishing PAAD immune landscapes, as well as drug candidates that could transform cold tumors into hot tumors.

## Materials and methods

### Data collection

RNA expression level data and clinical metadata were downloaded from The Cancer Genome Atlas (TCGA, https://www.cancer.gov/ccg/research/genome-sequencing/tcga), Cancer Genome Collaboratory (ICGC, https://dcc.icgc.org/) and Gene Expression Omnibus (GEO, https://www.ncbi.nlm.nih.gov/geo/) databases; data from normal tissues were excluded, and cancer sample data were retained. Seven datasets were included, namely, TCGA-PAAD, ICGC-PAAD-AU, ICGC-PAAD-CA, GSE85916 [20], GSE28735 [21], GSE62452 [22], and GSE78229 [23]. To assess gene expression in various cell types, eight PAAD single-cell RNA sequencing (scRNA-seq) datasets, including CRA001160 [24], GSE111672 [25], GSE141017 [26], GSE148673 [27], GSE154778 [28], GSE158356 [29], GSE162708 [30], and GSE165399 [31], were downloaded. Immunohistochemistry (IHC) data were downloaded from the Human Protein Atlas (HPA, http://www.proteinatlas.org) to verify protein expression in PAAD and normal tissues.

### Immune composition analysis and immune subtype clustering

The CIBERSORT algorithm [32] of the IOBR package (v.0.99.9) [33] was used to quantify the fractions of 22 immune cell types based on RNA expression data. The “ConsensusClusterPlus” package [34] was used to identify different clusters based on immune infiltration using a consensus clustering approach. The number of clusters was chosen according to the area under the cumulative distribution function (CDF) curve and the k values. To increase the reliability of our classification results, we repeated the classification step 1000 times. The “ESTIMATE” package in R (v 1.0.13) [35] was used to calculate the StromaScore, ImmuneScore, and EstimateScore values for each patient based on gene expression levels, which were used to define hot and cold clusters. The StromalScore quantifies the presence and extent of the stromal components within a tumor. The immuneScore evaluates the degree of immune cell infiltration within the TME. The ESTIMATEScore integrates the above aspects by estimating the proportions of stromal and immune cells in tumor samples using gene expression data.

### Differentially expressed gene (DEG) and miRNA analysis

The “voom” algorithm of the limma package was used to analyze DEGs between hot and cold tumors [36]. Raw read count data across samples were used as input for differential expression analysis. Genes with an adjusted *P* < 0.01 and a log fold change (logFC) greater than or less than one were identified as DEGs. miRNA expression data from patients with PAAD were obtained from the TCGA database. Correlation coefficient values were calculated between DEG and miRNA expression levels, and miRNAs with correlation coefficients > 0.7 were selected for further analysis.

### Functional pathway enrichment analysis

The "clusterProfiler" R package was used to perform Gene Ontology (GO) and Kyoto Encyclopedia of Genes and Genomes (KEGG) analyses of DEGs between hot and cold tumors [37]. Based on gene function, GO analysis yielded three categories: biological processes, cellular components, and molecular functions. Enriched GO terms and KEGG pathways were determined based on the criterion of adjusted *P* < 0.05. The gene set variation analysis (GSVA) package with default parameters [38] was used to identify distinct signature pathways between hot and cold tumors.

### Weighted correlation network analysis (WGCNA)

The WGCNA package was used to analyze coexpression gene networks in hot and cold tumors [39]. To identify modules significantly associated with hot and cold clusters, modules exhibiting correlation coefficient values > 0.3 were selected for further analysis.

### Cox regression analysis

A univariate Cox regression model in the survival (v 3.2–7) package was used to evaluate the prognostic significance of each gene based on survival time, survival status, and gene expression levels [40].

### Construction of a consensus prognostic model by ML

A consensus prognostic model for PAAD survival with high accuracy and stability was created by integrating 11 ML algorithms, including ANN, survival random forest (RF), lasso, Enet, supervised principal component, XGBoost, stepwise Cox, partial least squares regression Cox (plsRcox), gradient boosting decision tree, ridge, and survival support vector machines. All possible combinations of the direction parameters of the algorithms and the combined algorithms were computed to create separate optimal models for all patients with PAAD, patients with hot-tumor PAAD, and patients with cold-tumor PAAD.

### Predicting the importance of genes or drugs by ML

Personalized drugs that target a specific group of patients can be selected by studying therapeutic responses across different subgroups [15, 16]. The support SVM, ANN, Boruta, RF, and XGBOOST ML algorithms were used to predict the significance of individual genes for prognosis. The weights or importance values of genes were obtained from each algorithm. Subsequently, values were normalized, and mean values for individual genes were calculated from the five algorithms. Then, the significance of genes for predicting prognosis was assessed using the final values derived from the normalization of the z scores from the five algorithms.

### Drug prediction

Drug sensitivity data were obtained from the Genomics of Drug Sensitivity in Cancer database (https://www.cancerrxgene.org/) [41]. The R package oncoPredict (v 0.2) was used to predict the IC50 values of each drug [42]. Subsequently, correlations between drug sensitivity and the expression levels of selected genes were analyzed. Additionally, differences in drug sensitivity between patient subgroups were calculated.

### Predicting responses to immunotherapy

The tumor immune dysfunction and exclusion (TIDE) algorithm was used to model tumor immune evasion. Processed RNA expression level data from patients with PAAD were uploaded to the online TIDE database website (http://tide.dfci.harvard.edu/) to derive TIDE scores for each patient, predicting responsiveness to immunotherapy, with higher TIDE scores indicating decreased response rates to ICB treatment.

### Docking drugs and protein molecules

The software DOCK (v 6.10) was used to predict the binding patterns of small molecules and protein complexes. First, protein structures corresponding to identified genes were downloaded from the Protein Data Bank database (https://www.rcsb.org/) [43], and proteins were pretreated with UCSF Chimera (v 1.15) by adding hydrogen, assigning partial charges and protonation states, and minimizing energy [44]. Second, a subset of spheres was selected to represent the binding sites using the largest cluster generated by SPHGENs. Third, the chemical structures of the active drug compounds were collected from the ZINC15 database (https://zinc15.docking.org/) [45]. Finally, all compounds were docked into the binding sites of target proteins encoded by selected genes and visualized using UCSF chimera (v 1.14) and LigPlus (v 2019).

### Statistical analysis

R (v 4.0.5) software was used for data analysis and plotting. Correlations between two continuous variables were evaluated using Pearson's correlation coefficient. The chi-squared test was used to compare categorical variables, while the Wilcoxon rank-sum test or *t*-test was used to compare continuous variables.

## Results

### Immune profiling and consensus clustering of PAAD

**Fig. 1:**
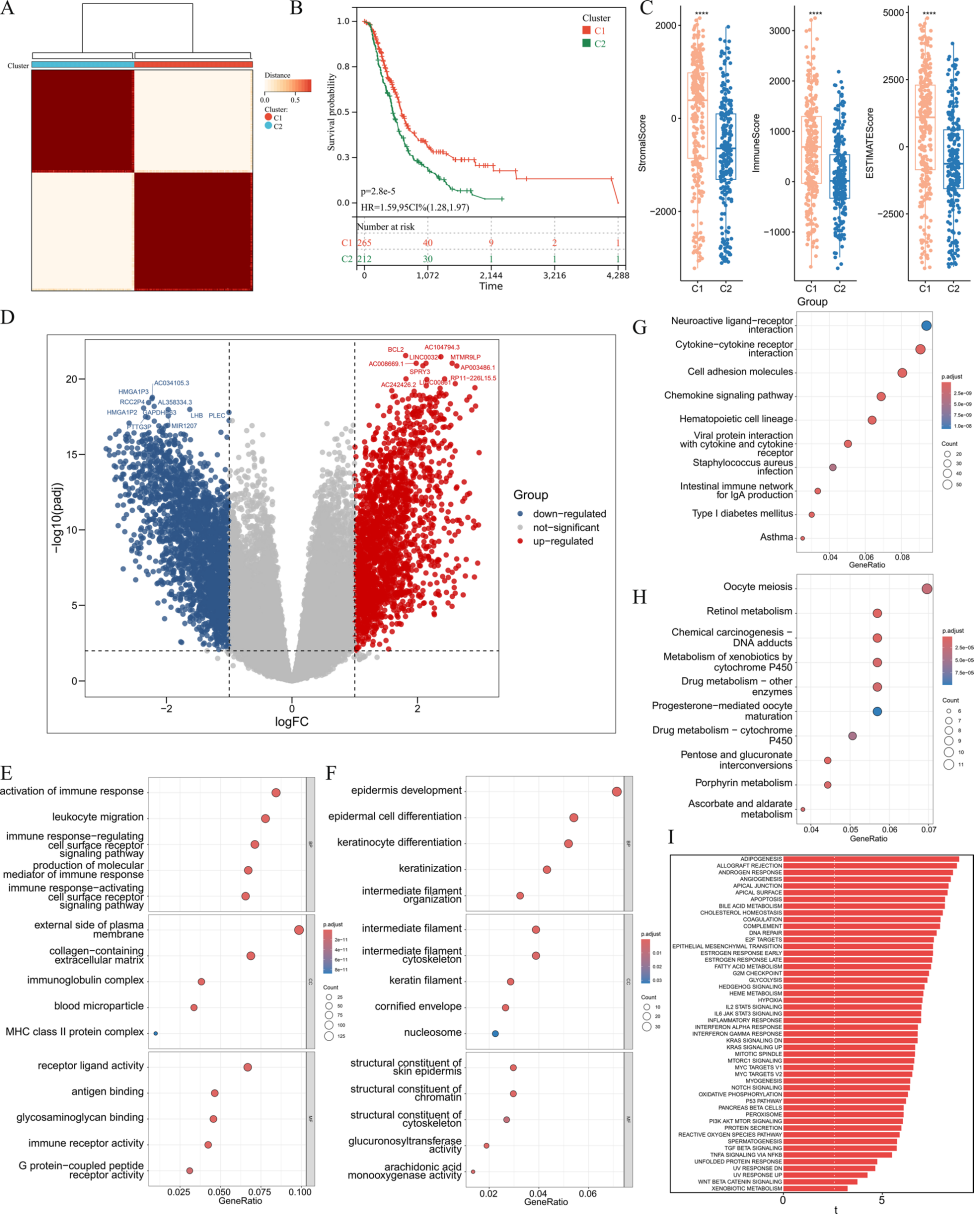
Identification of hot/cold tumors and DEGs **A** Validation of immune infiltration consensus clusters. **B** Comparison of survival probability between patients classified into the two clusters. **C** Comparisons of stromal, immune, and estimate scores between the two clusters. **D** DEGs between hot and cold tumors. Gene Ontology annotations of upregulated (**E**) and downregulated (**F**) genes. Kyoto Encyclopedia of Genes and Genomes pathways of upregulated (**G**) and downregulated (**H**) genes. **I** Gene set variation analysis showing hallmark pathways differing between hot and cold tumor samples

We combined TCGA-PAAD, ICGC-PAAD-AU, and ICGC-PAAD-CA data into a single database using the combat algorithm of the sva package [46] and removed batch effects. Next, we estimated the infiltration of 22 immune cell types based on the CIBERSORT algorithm and performed a consensus analysis using the ConsensusClusterPlus package according to the 22 immune cell fractions. Patients with PAAD were initially divided into 2–9 clusters, and CDF curves of the consensus score matrix and proportion of ambiguous clustering statistics were used to determine that the optimal number of clusters was 2 (Supplementary Fig. 1, Fig. 1A). Subsequent Cox analysis revealed a significant difference in survival between the two clusters (Fig. 1B). Based on the StromaScore, ImmuneScore, and EstimateScore, we defined cluster 1 tumors as “hot-immune” tumors and cluster 2 tumors as “cold-immune” tumors (Fig. 1C). Furthermore, we analyzed the differences in the infiltration of 22 immune cell types between hot and cold tumors; the proportions of naïve B cells, plasma cells, CD8^+^ T cells, resting memory CD4^+^ T cells, activated NK cells, monocytes, M1-type macrophages, M2-type macrophages, resting dendritic cells, and resting mast cells were greater in hot tumors (p < 0.001). In contrast, the frequencies of regulatory T cells (Tregs), resting NK cells, M0-type macrophages, and activated mast cells were greater in cold-treated tumors (p < 0.001) (Supplementary Fig. 2).

### Identification of DEGs and enriched pathways in hot versus cold tumors

We identified 2055 upregulated (Supplementary Table 1) and 2565 downregulated genes (Supplementary Table 2) in hot tumors relative to cold tumors using the “limma-voom” algorithm with the following criteria: “adj.P. Val” < 0.01 and abs of “logFC” > 1 (Fig. 1D, Supplementary Table 1). The clusterProfiler package was used to perform GO and KEGG analyses of the upregulated and downregulated genes, respectively. GO analysis elucidated that the pathways associated with immune responses and immune receptor activities were predominantly enriched in DEGs that were upregulated in hot tumors. These included pathways such as “activation of immune response”, “leukocyte migration”, “immune response-regulating cell surface receptor signaling pathway”, “antigen binding”, and “immune receptor activity” (Fig. 1E). Conversely, the pathways related to epidermis development, cytoskeleton, and chromatin structure were enriched in DEGs that were downregulated in hot tumors (Fig. 1F). KEGG analysis further indicated that the pathways related to immune ligand-receptor interactions were enriched in DEGs upregulated in hot tumors, such as “neuroactive ligand-receptor interaction”, “cytokine‒cytokine receptor interaction”, “cell adhesion molecules”, and “chemokine signaling pathway” (Fig. 1G), while some metabolism-related pathways were enriched in DEGs downregulated in hot tumors, including “retinol metabolism”, “chemical carcinogenesis DNA adducts”, “drug metabolism”, and “porphyrin metabolism” (Fig. 1H). We also performed GSVA and identified multiple hallmark pathways enriched in hot tumors (Fig. 1I), among which the top five were “adipogenesis”, “allograft rejection”, “androgen response”, “angiogenesis”, and “apical junction” (Fig. 1I).

### Identification of prognostic and immune-related gene signatures

**Fig. 2:**
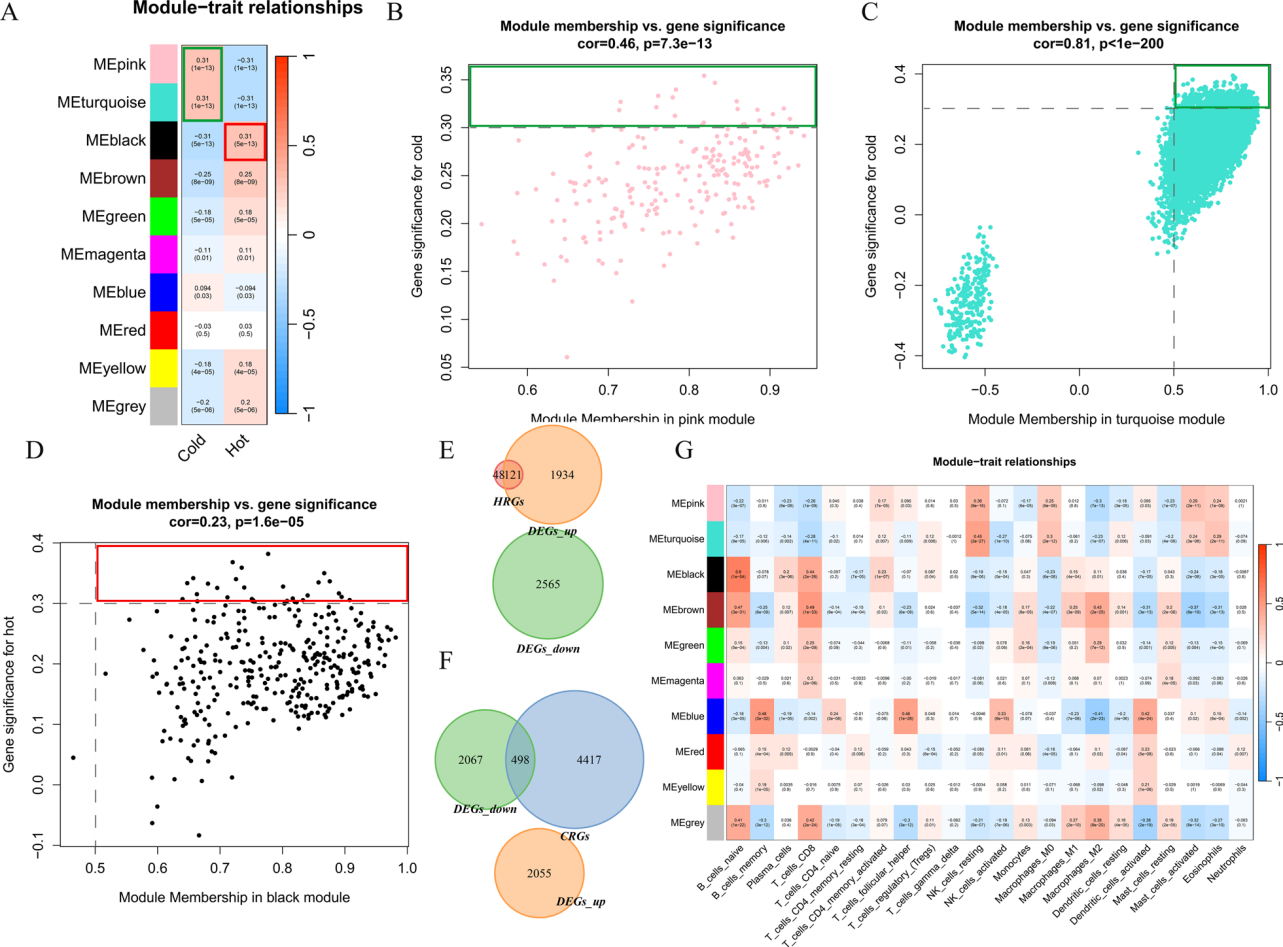
Weighted correlation network analysis (WGCNA). **A** WGCNA procedure and correlations between modules and hot/cold traits. Correlation coefficients between gene significance (GS) and module membership (MM) in the **B** pink, **C** turquoise, and **D** black modules. **E**, **F** Venn diagrams showing immune-related genes and DEGs. **G** Correlations between modules and immune cells

Ten modules indicated by different colors were identified using the WGCNA procedure. We computed the correlations between each module and immune clusters or immune cells and found that the pink and turquoise modules were related to cold tumors, while the black module was related to hot tumors, both with correlation coefficient values > 0.3 (Fig. 2A). In the pink module, the correlation coefficient between gene significance (GS) and module membership (MM) was 0.46 (Fig. 2B), while the correlation coefficients for the turquoise and black modules were 0.81 and 0.23, respectively (Fig. 2C and D).

Genes with GS > 0.3 and MM > 0.5 were selected for further analysis (Fig. 2B–D), which included 165 hot-related genes (HRGs) and 4183 cold-related genes (CRGs) (Fig. 2E, F). The intersection of the DEGs and WGCNA results revealed 118 genes that overlapped among the upregulated DEGs and HRGs and 375 genes that overlapped among the downregulated DEGs and CRGs; these genes were extracted for subsequent analysis (Fig. 2E, F). Cox regression analysis based on survival time, survival status, and gene expression was performed to assess prognostic significance. We found that 82 genes upregulated in hot tumors had prognostic significance and were immune-related; these genes were designated 'upregulated in hot tumors, prognostic, and immune-related genes' (UPIRGs). Furthermore, 96 genes that were downregulated in hot tumors, had prognostic significance and were immune-related; these genes are referred to as 'Downregulated in hot tumors, Prognostic, and Immune-Related Genes' (DPIRGs) (P < 0.05) (Supplemental Figs. 3A, B).

**Fig. 3:**
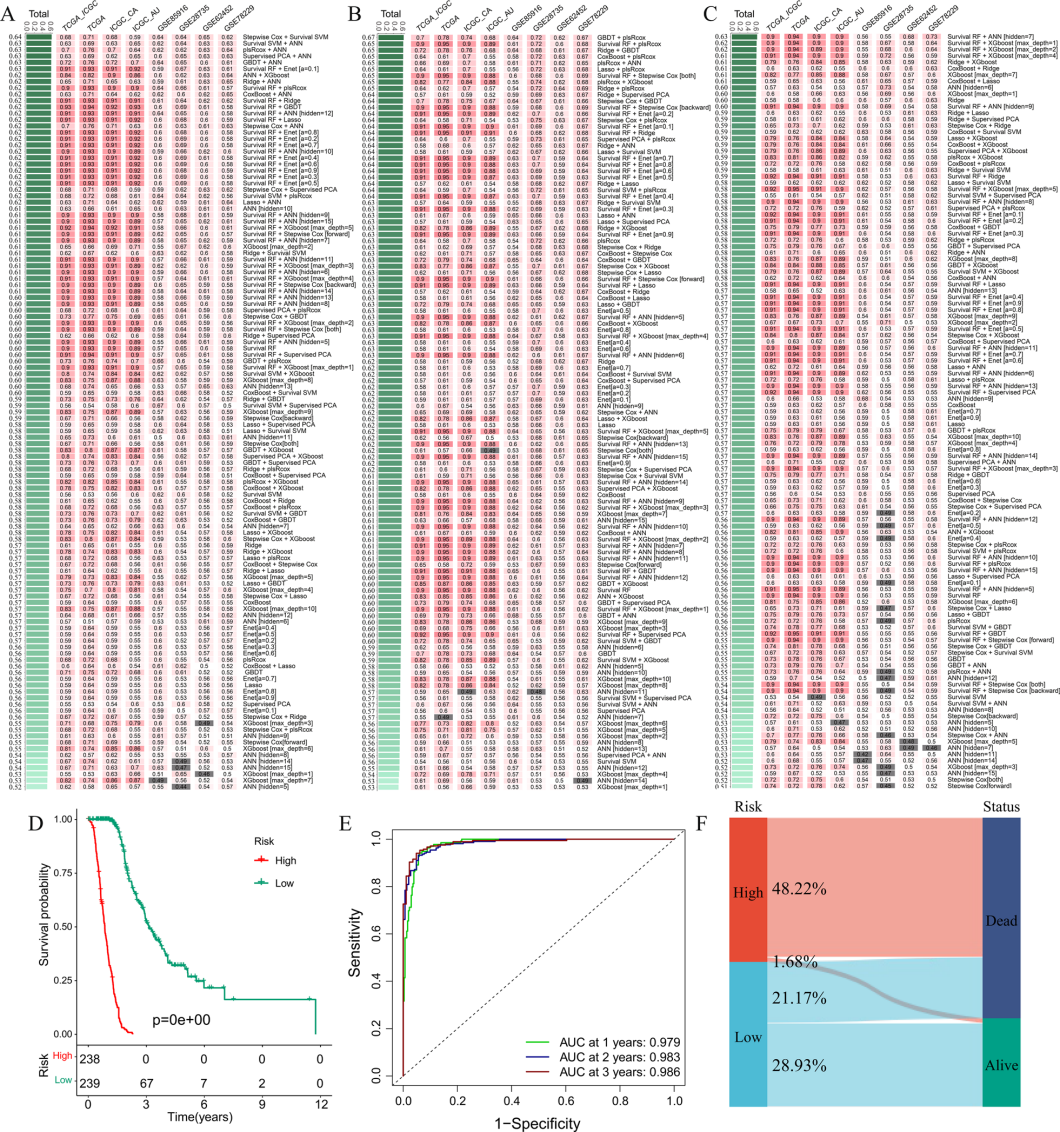
Construction and validation of prognostic signatures. C-index values of the training and validation prognostic signature sets, based on ‘upregulated in hot tumors, prognostic, and immune-related genes’ (UPIRGs) (**A**), ‘downregulated in hot tumors, prognostic, and immune-related genes’ (DPIRGs) (**B**), and both UPIRGs and DPIRGs (**C**) for each model. **D** Survival curves of patients in the high- and low-risk groups among the mixed group of patients with pancreatic adenocarcinoma. **E** ROC curves show the AUC values of the model for different survival times. **F** Sankey plot showing the proportions of surviving and deceased patients in the high- and low-risk groups

In addition, correlation analysis indicated that genes in the pink and turquoise modules may negatively regulate CD8^+^ T cells, M2-type macrophages, and resting mast cells (R < − 0.2) and positively regulate resting NK cells, M0-type macrophages, activated mast cells, and eosinophils (R > 0.2). Genes in the black module were predicted to upregulate naïve B cells, CD8^+^ T cells, and memory-resting CD4^+^ T cells (R > 0.2) (Fig. 2G) but negatively regulate M0-type macrophages and activated mast cells (R < − 0.2) (Fig. 2G).

### Construction of a consensus ML-driven prognostic model

To develop a consensus model, 82 UPIRGs, 96 DPIRGs, and all 178 prognostic immune-related genes were subjected to our ML-based integrative procedure using the leave-one-out cross-validation (LOOCV) framework. In the mixed TCGA + ICGC training cohort, we constructed models based on single and combined ML algorithms and computed C-index values for each model. Furthermore, we calculated the C-index values for the training model using the TCGA-PAAD, ICGC-CA, and ICGC-AU datasets and then calculated the mean C-index across the four GSE datasets to assess the predictive power of all the models (Fig. 3A–C). The results showed that the optimal model for UPIRGs was a combination of Survival RF and Enet (a = 0.1) (Fig. 3A); for DPIRGs, it was a combination of Survival RF and PlsRcox (Fig. 3B); and for PIRGs, it was a combination of Survival RF and ANN (hidden = 7) (Fig. 3C). Based on these three models, a model combining Survival RF and PlsRcox for DPIRGs was found to be the optimal prognostic model with the best C-index and was designated the mixed model.

To assess the prognostic significance of DPIRGs, we divided patients with PAAD into high- and low-risk groups based on the median value of the risk score. According to the TCGA + ICGC training database, there was a significant difference in survival between the high- and low-risk groups (p < 0.001), with the low-risk group having a better survival probability (Fig. 3D). Consistently, the area under the curve (AUC) values of the time-dependent ROC curves for 1-, 2-, and 3-year survival in the TCGA + ICGC cohort were 0.979, 0.983 and 0.986, respectively (Fig. 3E). We also compared the risk scores and clinical status between the two groups and demonstrated that the high-risk group had a greater mortality rate than the low-risk group (Supplementary Figs. 4A, B). The high-risk group had a lower survival rate (Fig. 3F).

**Fig. 4:**
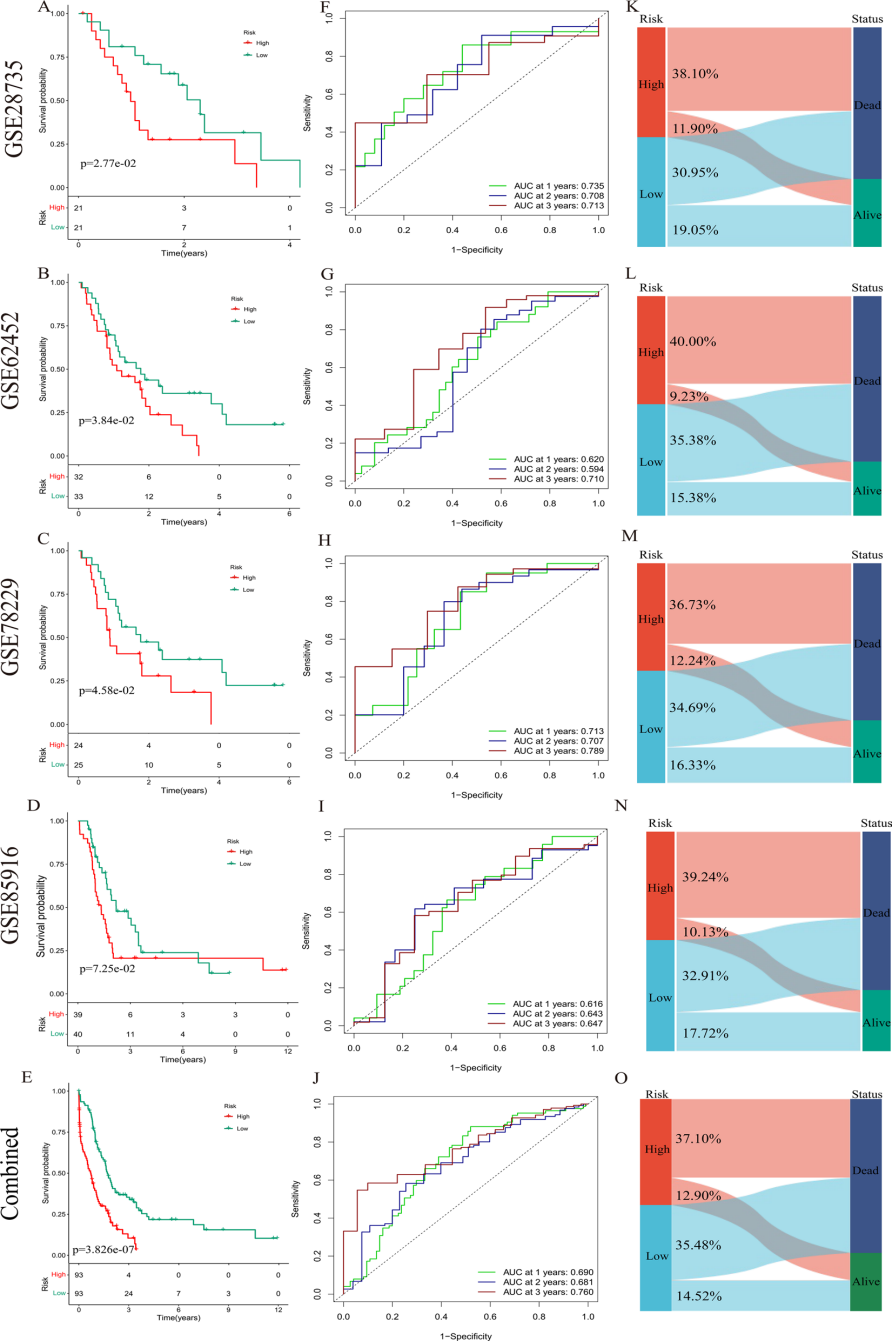
Survival curves, ROC curves, and Sankey plots show the proportions of surviving and deceased patients in the four GSE datasets. **A**, **F**, and **K** GSE28735. **B**, **G**, and **L** GSE62452. **C**, **H**, and **M** GSE78229. **D**, **I**, and **N** GSE85916. **E**, **J**, and **O** Combined validation database

Next, four GSE datasets were used to validate the feasibility of using DPIRGs to predict prognosis in patients with PAAD. We also combined the four validation datasets to form a combined test. K‒M survival curves showed that patients in the low-risk group had better overall survival (OS) than those in the high-risk group (Fig. 4A–E). The AUC values for the ROC curves were 0.735 for GSE28735, 0.710 for GSE62452, 0.789 for GSE78229, 0.647 for GSE85916, and 0.760 for the combined test (Fig. 4F–J). We also compared risk scores and clinical status between the two groups in the four GSE datasets (Supplementary Figs. 4C–J), and our model showed that more patients in the high-risk groups died than in the respective low-risk groups (Fig. 4K–O). Overall, KM survival analysis, time-dependent ROC curve analysis, and calculation of C-index values for one training cohort and four validation cohorts consistently indicated that the DPIRG signature could potentially predict the outcomes of patients with PAAD in the external validation cohorts.

### Prognostic value of the DPIRG signature in PAAD

**Fig. 5:**
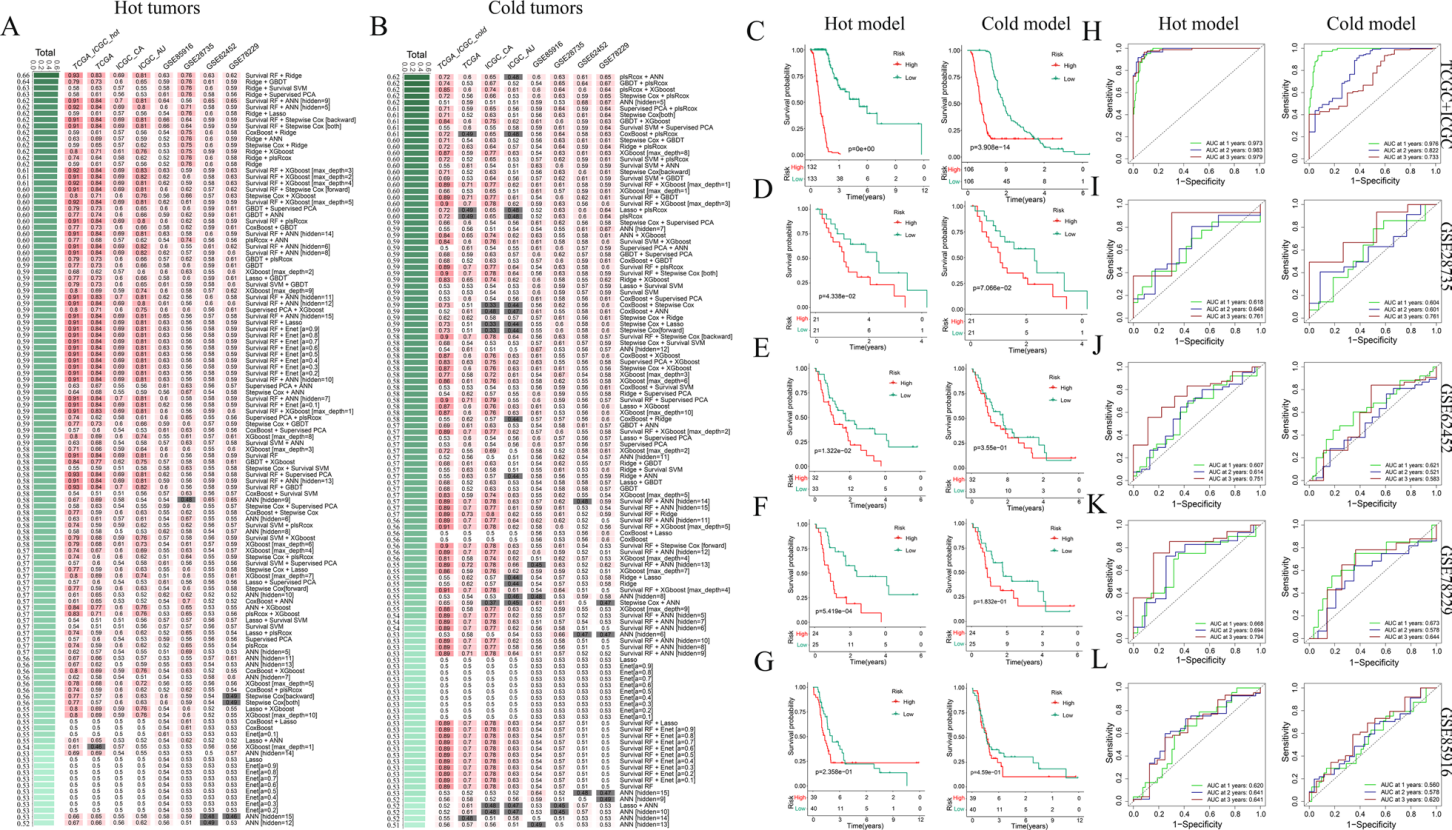
Construction and validation of prognostic signatures of patients with hot/cold pancreatic adenocarcinoma (PAAD) tumors. C-index values of the test and validation prognostic signature sets for **A** hot and **B** cold tumors, based on ‘Downregulated in hot tumors, Prognostic, and Immune-Related Genes’ for each model. Survival curves of PAAD patients with hot (left) and cold (right) tumors in the **C** TCGA + ICGC, **D** GSE28735, **E** GSE62452, **F** GSE78229, and **G** GSE85916 datasets based on hot- and cold-tumor prognostic signature models. ROC curves showing AUC values of the model for different survival times in PAAD patients with hot (left) and cold (right) tumors in the **H** TCGA + ICGC, **I** GSE28735, **J** GSE62452, **K** GSE78229, and **L** GSE85916 datasets based on hot- and cold-tumor prognostic signature models

After constructing a prognostic model for patients with PAAD based on ML, we also examined whether distinct prognostic models could be applied separately to those with hot and cold tumors. Therefore, single and combined ML algorithms were used to construct consensus models for patients with hot or cold tumors based on DPIRGs. According to the mean C-index values across the four GSE validation datasets for all the models, the optimal model for patients with hot tumors was a combination of survival RF and ridge, which we designated the hot model (Fig. 5A), and the optimal model for those with cold tumors was a combination of plsRcox and XGBoost, which we designated the cold model (Fig. 5B). Cox analysis showed that the hot model was better for dividing patients into high- and low-risk groups than the cold model; differences in survival between the risk groups were more significant according to the hot model and data from the TCGA + ICGC and the four GSE databases (Fig. 5C–G).

Furthermore, we compared the mixed model to the hot model. We found that only the GSE78229 dataset had a greater difference in survival according to the hot model (Fig. 5F, left) than according to the mixed model (Fig. 4C). In contrast, more significant differences in survival were generated for the other databases using the mixed model (Fig. 4A, B, D). Next, we focused on validating the abilities of the mixed, hot, and cold models in predicting PAAD prognosis using data from the training database and four GSE databases. We found that the mixed (Fig. 4F–I) and hot models (Fig. 5I–L, left) performed better, with higher AUC values, than did the cold model (Fig. 5I–L, right). In conclusion, the mixed and hot models had advantages in different datasets, while the cold model performed less well in prognosis prediction.

### Analysis of genetic alterations and DNA methylation of DPIRGs

**Fig. 6 ‘:**
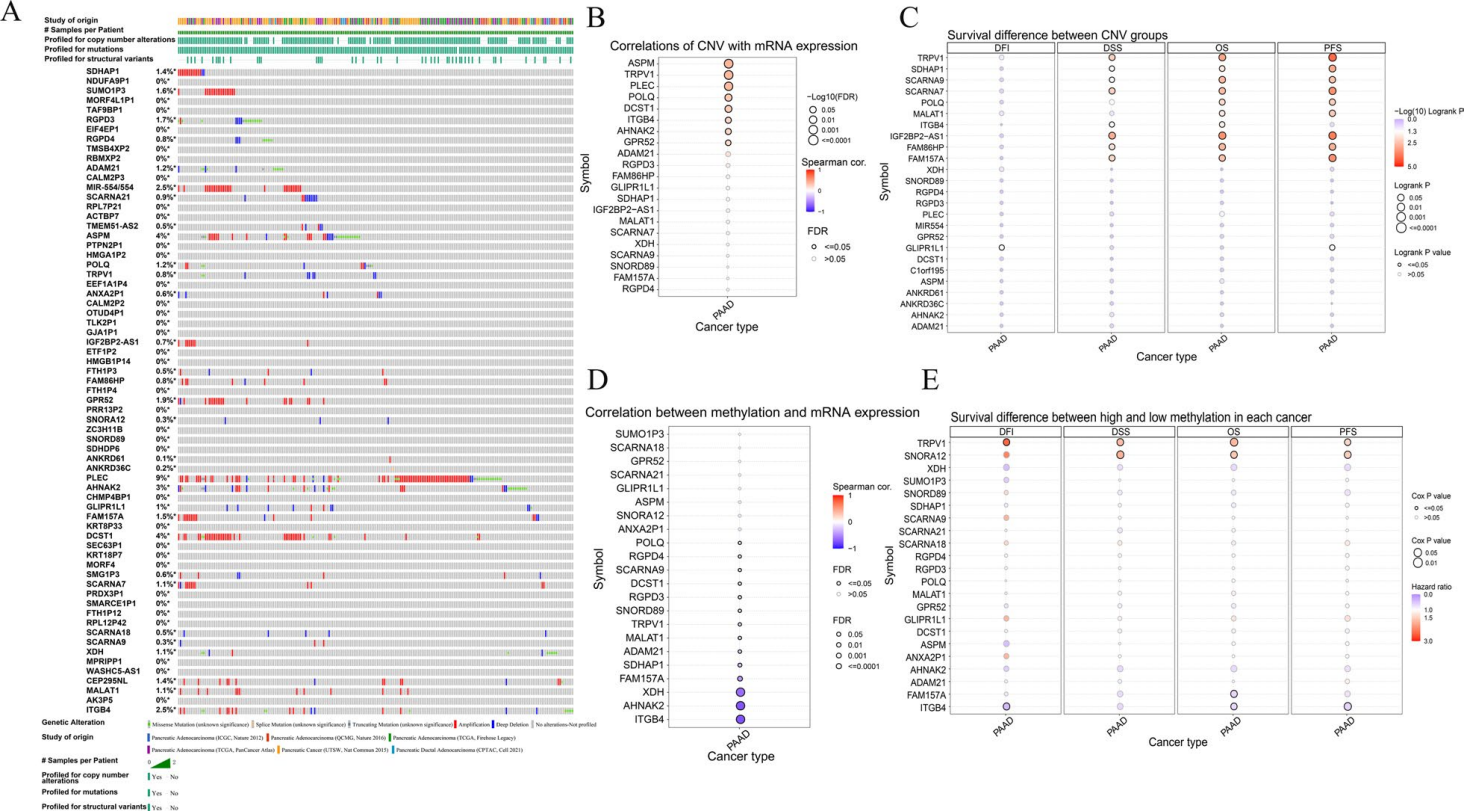
Downregulated in hot tumors, Prognostic, and Immune-Related Gene’ (DPIRG) mutations. **A** Waterfall plots showing the frequencies of mutations and copy number variations (CNVs) of DPIRGs. **B** Correlations between CNVs and RNA expression of DPIRGs. **C** Comparison of survival between patients with and without CNVs in DPIRGs. **D** Correlation between DNA methylation and RNA expression of DPIRGs. **E** Differences in survival between patients with high and low DPIRG methylation status

Our findings demonstrate that the DPIRG signature is significantly associated with the prognosis of patients with PAAD. To explore the mechanisms that regulate DPIRG expression, we used cBioPortal (http://www.cbioportal.org/) to elucidate the genetic alterations of DPIRGs in PAAD. Some gene mutations were identified; however, the mutation frequencies were relatively low (for example, *ASPM*, 4%; *AHNAK2*, 3%; and *DCST1*, 4%), with *PLEC* having the highest mutation frequency of 9% (Fig. 6A). We also evaluated the relationship between gene copy number variation (CNV) and expression level. Positive correlations between CNVs and *ASPM*, *TRPV1*, *PLEC*, *POLQ*, *DCST1*, *ITGB4*, *AHNAK2*, and *GPR52* expression levels were detected (Fig. 6B). Furthermore, survival analysis of patients with CNVs of these DPIRGs was performed, and the results indicated that CNVs of *TRPV1, SDHAP1*, *SCARNA9*, *SCARNA7*, *IGF2BP2-AS1*, *FAM86HP*, and *FAM157A* were significantly associated with disease-specific survival (DSS), progression-free survival (PFS), and OS (Fig. 6C).

Next, the effect of DNA methylation on the expression of these DPIRGs was explored, and the results indicated that the DNA methylation levels of *ITGB4, AHNAK2*, and *XDH* were negatively correlated with their expression (Fig. 6D). We also found that in patients with PAAD, *TRPV1* DNA methylation was associated with disease-free interval (DFI), DSS, PFS, and OS (Fig. 6E); *SNORA12* DNA methylation was related to PFS, PFS, and OS; and *ITGB4* DNA methylation was associated with DFI, PFS, and OS (Fig. 6E).

### Predicting DPIRGs is valuable for PAAD prognosis and clustering by ML.

**Fig. 7:**
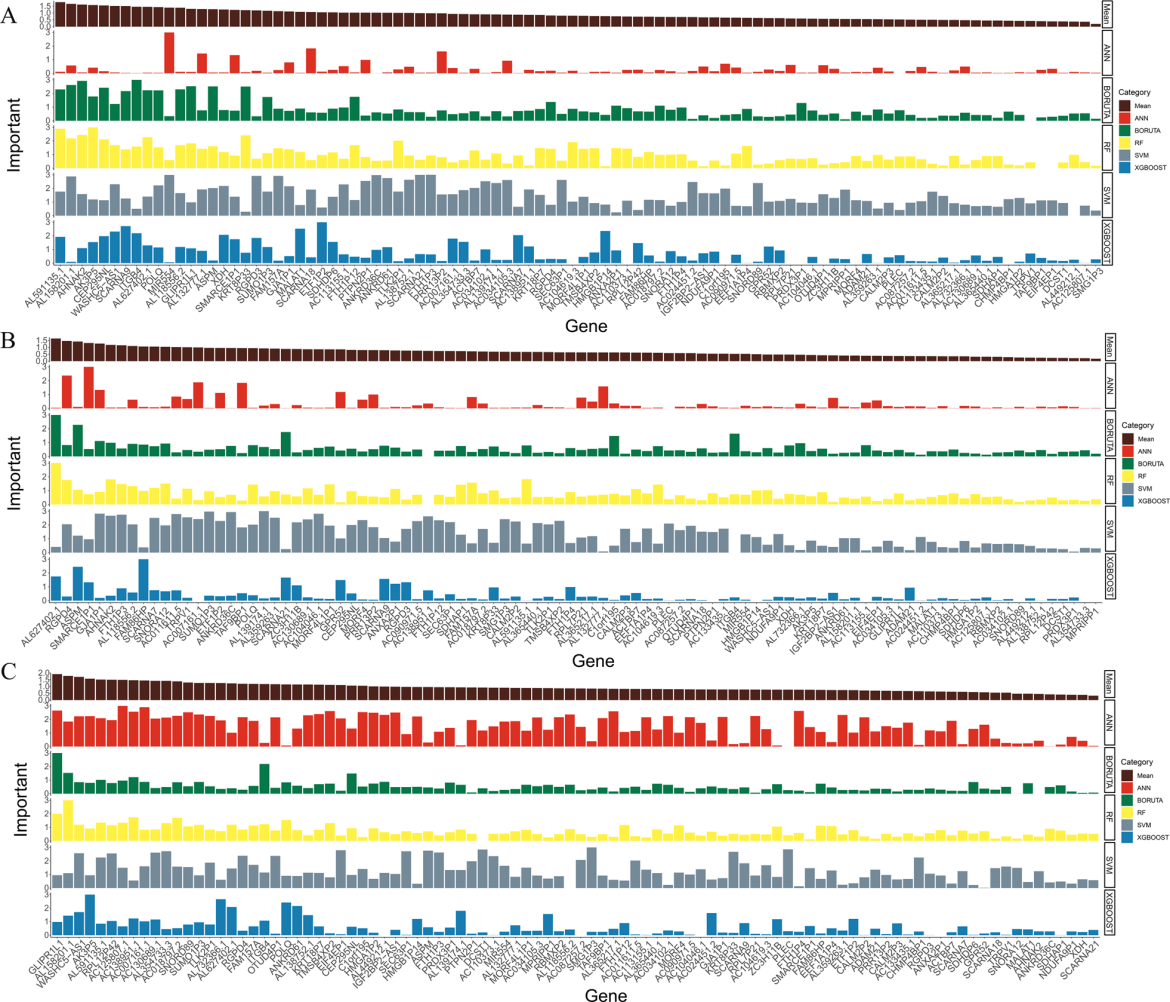
The ability of ‘downregulated in hot tumors’, ‘prognostic’, and ‘immune-related genes’ (DPIRGs) to predict prognosis. **A** Prediction of the importance of DPIRGs for predicting the prognosis of patients with pancreatic adenocarcinoma in the mixed (**A**), hot-tumor (**B**), and cold-tumor (**C**) groups using five machine learning algorithms

To understand the potential biological functions and mechanisms by which DPIRGs influence the tumor immune landscape and prognosis of PAAD patients, we first evaluated the significance of DPIRGs with five ML algorithms, namely, SVM, ANN, Boruta, RF, and XGBOOST, in mixed, hot, and cold tumors. In mixed tumors, the values of the DPIRGs were normalized for each ML algorithm (Supplementary Fig. 5), and the top 5 genes most significantly associated with patient survival were identified as *AL591135.1*, *AL158201.1*, *AHNAK2*, *AK3P5*, and *CEP295NL* (Fig. 7A, Supplementary Fig. 5). The top 5 genes in hot tumors were *AL627402.1*, *RGPD4*, *ASPM*, *SMARCE1P1*, and *GJA1P1* (Fig. 7B, Supplementary Fig. 6), and the top 5 genes in cold tumors were *GLIPR1L1*, *AL158201.1*, *WASHC5-AS1*, *AK3P5*, and *AL591135.1* (Fig. 7C, Supplementary Fig. 7). In addition, ROC analysis indicated that the top 10 genes, namely, *HMGA1P2*, *C1orf195*, *ITGB4*, *AC087257.2*, *AC011611.5*, *AC034105.3*, *AC110373.1*, *AL449212.1*, *AL354733.3*, and *DCST1*, could effectively distinguish patients with hot and cold PAAD tumors, with AUC values ranging from 0.715 to 0.728 (Supplementary Fig. 8).

**Fig. 8:**
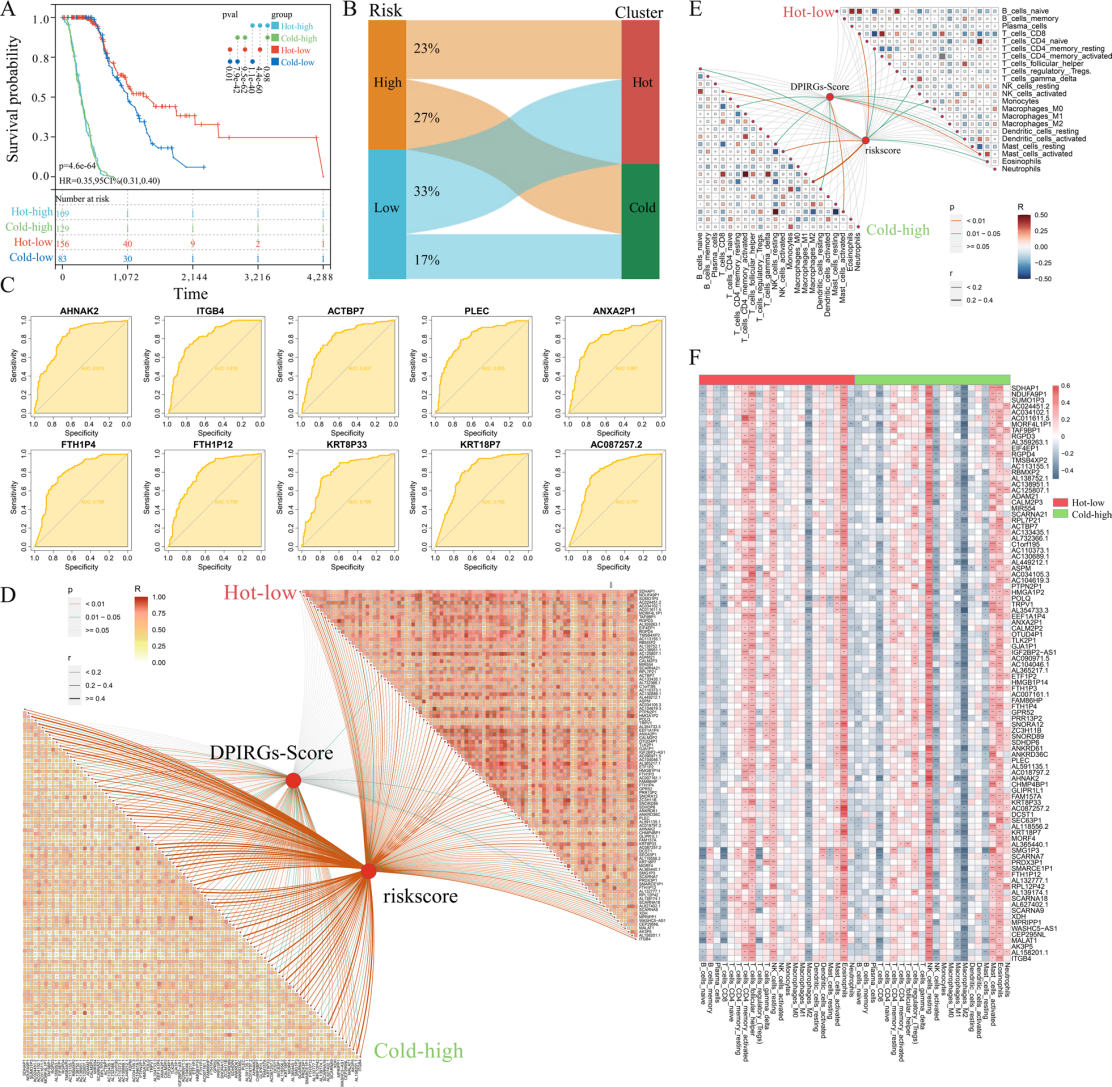
Correlations between genes and between immune cells. **A** Survival curves of patients with pancreatic adenocarcinoma in the hot-high, cold-high, hot-low, and cold-low groups. **B** Sankey plot showing the proportions of patients with high/low risk and hot/cold tumors. **C** ROC curves predicting hot-low and cold-high tumors with the 10 genes with the highest AUC values. **D** Correlations between ‘downregulated in hot tumors’, ‘prognostic’, and ‘immune-related genes’ (DPIRGs) in the hot-low and cold-high groups. **E** Correlations between immune cells in the hot-low and cold-high groups. (F) Correlations between DPIRGs and immune cells in the hot-low and cold-high groups (ns: p > 0.05, *p < 0.05, **p < 0.01, ***p < 0.001, ****p < 0.0001)

Patients with PAAD were divided into four groups based on immune subtypes and risk levels: hot-high, hot-low, cold-high, and cold-low. The hot-low group had the best prognosis among the four groups, whereas the hot-high and cold-high groups had the worst prognosis (Fig. 8A). According to the mixed database, more patients were assigned to the hot-low group than to the cold-low group (33% vs. 17%), suggesting that low-risk patients tended to have hot tumors. In contrast, high-risk patients were evenly distributed into hot and cold groups (23% vs. 27%, respectively) (Fig. 8B). Furthermore, we conducted ROC analysis to predict the survival of patients with hot-low and cold-high tumors and found that the top 10 genes that could effectively distinguish hot-low and cold-high tumors were *AHNAK2*, *ITGB4*, *ACTBP7*, *PLEC*, *ANXA2P1*, *FTH1P4*, *FTH1P12*, *KRT8P33*, *KRT18P7*, and *AC087257.2*, with AUCs ranging from 0.816 to 0.791 (Fig. 8C, Supplementary Fig. 9).

### Associations of DPIRGs with PAAD risk subtypes

To examine potential interactions between individual DPIRGs, we estimated correlations between individual DPIRGs and found that overall correlations among DPIRG expression levels were greater in the hot-low group than in the cold-high group (Fig. 8D, Supplementary Tables 3, 4). Next, to examine the associations of individual DPIRGs with different PAAD risk groups, we analyzed correlations between individual DPIRG expression levels and DPIRG scores calculated using the ssGSVA algorithm (Supplementary Tables 5–6). Overall, the correlations between DPIRG expression and DPIRG score were greater in the cold-high group than in the hot-low group. Similarly, correlations between individual DPIRG expression levels and risk scores were greater in the cold-high group (Fig. 8D, Supplementary Tables 7, 8), suggesting that DPIRGs may more significantly influence the PAAD risk level in the cold-high group.

### Roles of DPIRGs in shaping the immune landscape of PAAD subtypes

To better understand the immune landscapes of different PAAD subgroups, we first calculated the correlations between multiple immune cell types to evaluate their potential interactions. The results indicated that naïve B cells were positively correlated with plasma cells (R = 0.418), CD8 + T cells (R = 0.403), and Tregs (R = 0.403) and negatively correlated with memory-activated CD4^+^ T cells (R = − 0.378) in the cold-high group, while in the hot-low group, their correlation coefficients were weaker (Fig. 8E, Supplementary Tables 9, 10). In addition, CD8^+^ T cells were positively correlated with memory-activated CD4^+^ T cells (R = 0.412) and M1-type macrophages (R = 0.306), while they were negatively correlated with memory-resting CD4^+^ T cells (R = − 0.378) and activated mast cells (R = − 0.342) in the cold-high group (Fig. 8E, Supplementary Tables 9, 10). In the hot-low group, CD8^+^ T cells were positively correlated with M1-type macrophages (R = 0.371), while they were negatively correlated with memory-resting CD4^+^ T cells (R = − 0.370) and activated mast cells (R = − 0.370) (Fig. 8E, Supplementary Tables 9, 10). In the cold-high group, the highest correlation coefficient was between M1-type macrophages and memory-activated CD4^+^ T cells (R = 0.495), and the lowest was between activated mast cells and resting mast cells (R = − 0.489); in the hot-low group, the highest correlation coefficient was also between M1-type macrophages and memory-activated CD4^+^ T cells (R = 0.509), and the lowest was between memory resting CD4^+^ T cells and CD8^+^ T cells (R = − 0.438) (Fig. 8E, Supplementary Tables 9, 10).

We also estimated the correlations between various immune cells and the DPIRG score (Supplementary Tables 11, 12) and between immune cells and the risk score (Supplementary Tables 13, 14). M2-type macrophages were positively correlated with risk scores in the cold-high group (Fig. 8E), whereas their correlation was not significant in the hot-low group. By calculating the correlations between genes and immune cells, we identified more genes that may positively regulate resting NK cells and activated mast cells and negatively regulate M2-type macrophages, CD8^+^ T cells, and activated NK cells (p < 0.05) in the cold-high group (Fig. 8F), whereas more genes that could positively regulate memory-activated CD4^+^ T cells, follicular helper T cells, and eosinophils (p < 0.05) were identified in the hot-low group (Fig. 8F). Furthermore, the StromaScore, ImmuneScore, and EstimateScore values were negatively correlated with the RNA expression levels of DPIRGs in the hot-low and cold-high groups (Supplementary Fig. 10).

### miRNA and GSVA analysis of PAAD subtypes

**Fig. 9:**
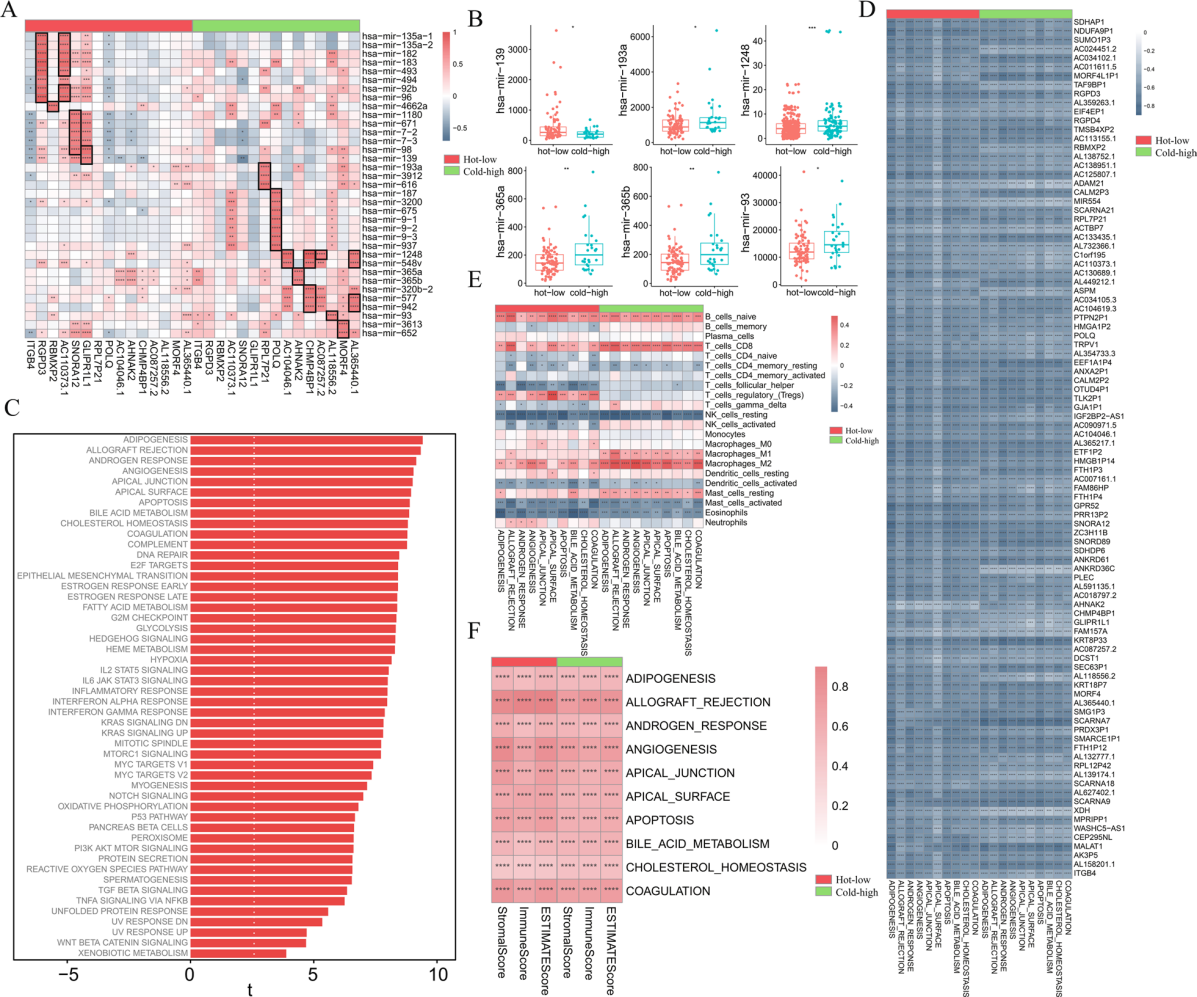
miRNA coexpression and gene set variation analysis (GSVA). **A** Correlations between miRNAs and downregulated in hot tumors, prognostic genes, and immune-related genes (DPIRGs) in the hot-low vs. cold-high groups. **B** Differences in the expression of miRNAs between the hot-low and cold-high groups. **C** Hallmark pathways differ between the hot-low and cold-high groups based on GSVA. Correlations between DPIRGs (**D**), immune cells (**E**), and three immune-related scores (**F**) and the top 10 hallmark pathways in the hot-low vs. cold-high groups (ns: p > 0.05, *p < 0.05, **p < 0.01, ***p < 0.001, ****p < 0.0001)

To explore whether miRNAs are involved in the function of DPIRGs, we searched for miRNAs whose levels were significantly associated with the expression of DPIRGs (correlation coefficient > 0.7) and identified 16 miRNAs in the hot-low group and 20 miRNAs in the cold-high group (Fig. 9A). We then evaluated differences in the expression of these 36 miRNAs and found that the expression of only hsa-mir-139 was greater in the hot-low group, while the expression levels of hsa-mir-193a, hsa-mir-1248, hsa-mir-365a, hsa-mir-365b, and hsa-mir-93 were greater in the cold-high group.

To explore whether DPIRGs exert different biological functions in hot-low and cold-high PAAD tumors, we conducted GSVA; the hallmark pathways identified as enriched in the hot-low group relative to the cold-high group are shown in Fig. 9C. In both groups, naïve B cells were positively correlated with the top 10 enriched hallmark pathways, whereas resting NK cells, activated mast cells, and eosinophils were negatively correlated with these pathways (Fig. 9E). In general, CD8^+^ T cells, M1 and M2 macrophages, and resting mast cells were more significantly correlated with pathways in the cold-high group, while Tregs, follicular helper T cells, and activated dendritic cells were more significantly correlated with pathways in the hot-low group (Fig. 9E). Interactions between these biological pathways and various immune cells may be involved in determining the outcomes of PAAD subtypes.

### Immunotherapeutic response analysis and drug prediction

**Fig. 10:**
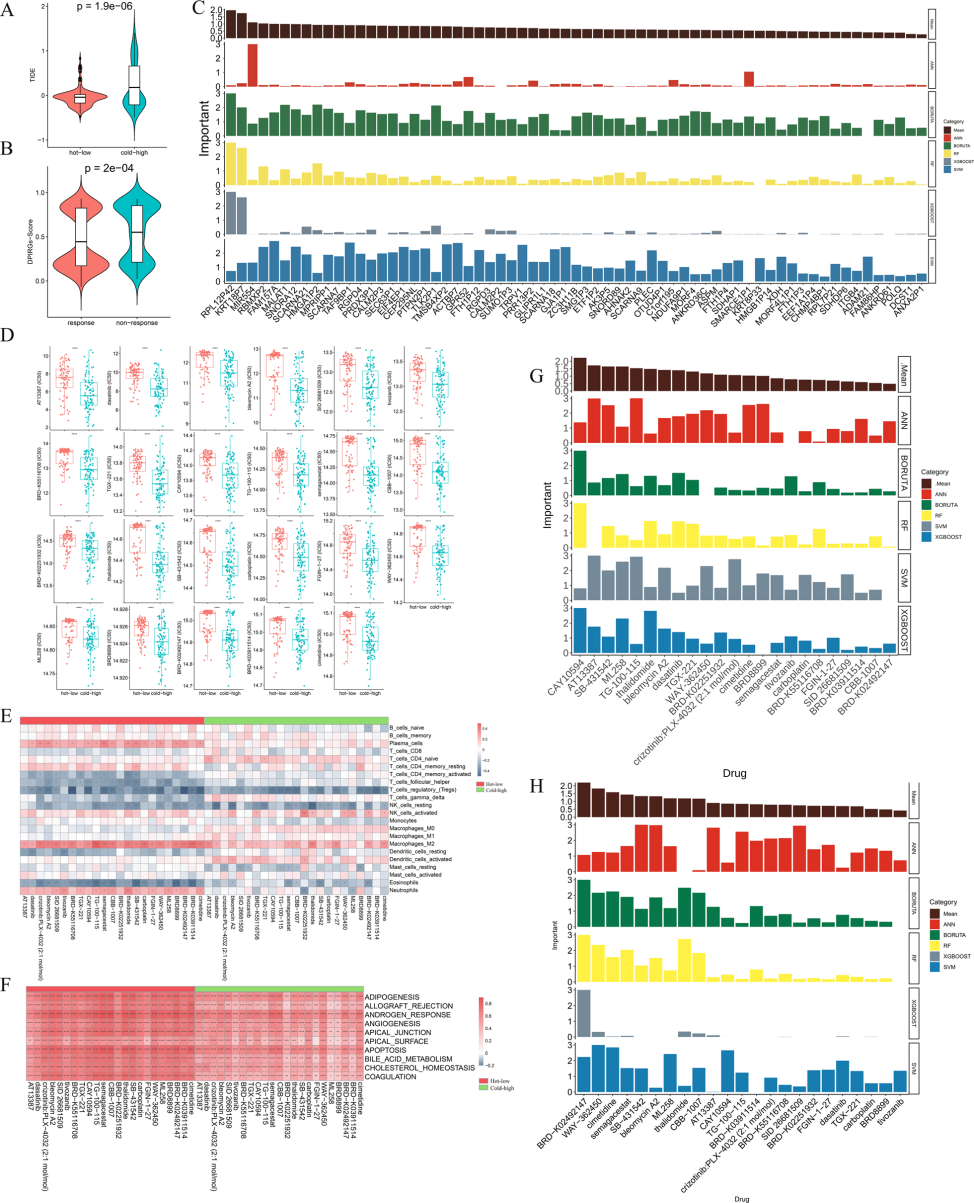
Immunotherapy and drug prediction. **A** Comparison of TIDE scores between the hot-low and cold-high groups. **B** Comparison of ‘Downregulated in hot tumors, Prognostic, and Immune-Related Gene’ (DPIRG) scores between patients in the PD-1 blockade responder and nonresponder groups. **C** Prediction of the importance of DPIRGs for TIDE scores using five machine learning algorithms. **D** Comparison of the sensitivity to drugs in the hot-low and cold-high groups. Correlations between immune cells (**E**), the top 10 hallmark pathways (**F**), and predicted drug responses in the hot-low and cold-high groups. Prediction of the importance of predicted drugs for the prognosis of patients with pancreatic adenocarcinoma (**G**) and DPIRG score (**H**) using five machine learning algorithms (ns: p > 0.05, *p < 0.05, **p < 0.01, ***p < 0.001, ****p < 0.0001)

The TIDE score is closely correlated with the potential for tumor immune escape and resistance to immunotherapy, with higher TIDE scores predicting lower immunotherapy response rates. As shown in Fig. 10A, TIDE scores in hot-low tumors were significantly lower than those in cold-high tumors. In addition, DPIRG scores were significantly lower in patients who responded to PD-1 blockade than in nonresponders (Fig. 10B). We also predicted significant DPIRGs that affected TIDE scores using five ML algorithms and found that the top 5 genes were *RPL1P42*, *KRT18P7*, *MIR554*, *RBMXP2*, and *FAM157A* (Fig. 10C).

Next, we used the OncoPredict package to calculate the correlation between DPIRG expression and the existing drug response. Twenty-three drugs were negatively associated with DPIRG expression levels (Supplementary Fig. 11, Supplementary Tables 15, 16), and the IC50 values of these 23 drugs differed between the hot-low and cold-high groups, with higher values in the hot group, indicating that these drugs may have more potent therapeutic effects in the cold-high group (Fig. 10D). In the hot-low group, the 23 drugs positively regulated plasma cells, M2-type macrophages, and neutrophils, while they negatively regulated follicular helper T cells and eosinophils. In the cold-high group, the drugs were strongly negatively associated with Tregs and resting NK cells (Fig. 10E). Additionally, these drugs were positively associated with hallmark pathways, with higher correlation coefficients in the hot-low group than in the cold-high group (Fig. 10F).

Next, five ML algorithms were used to predict the significance of drugs for prognosis, and the top 8 drugs associated with patient survival were CAY10594, thalidomide, AT13387, SB-431542, dasatinib, bleomycin A2, ML258, and TGX-221 (Fig. 10G). We also used five ML algorithms to predict drugs that could significantly regulate the DPIRG score, and the top 8 drugs were BRD-K02251932, WAY-362450, cimetidine, semagacestat, SB-431542, bleomycin A2, ML258, and thalidomide (Fig. 10H).

### Binding of drug molecules to DPIRGs

**Fig. 11:**
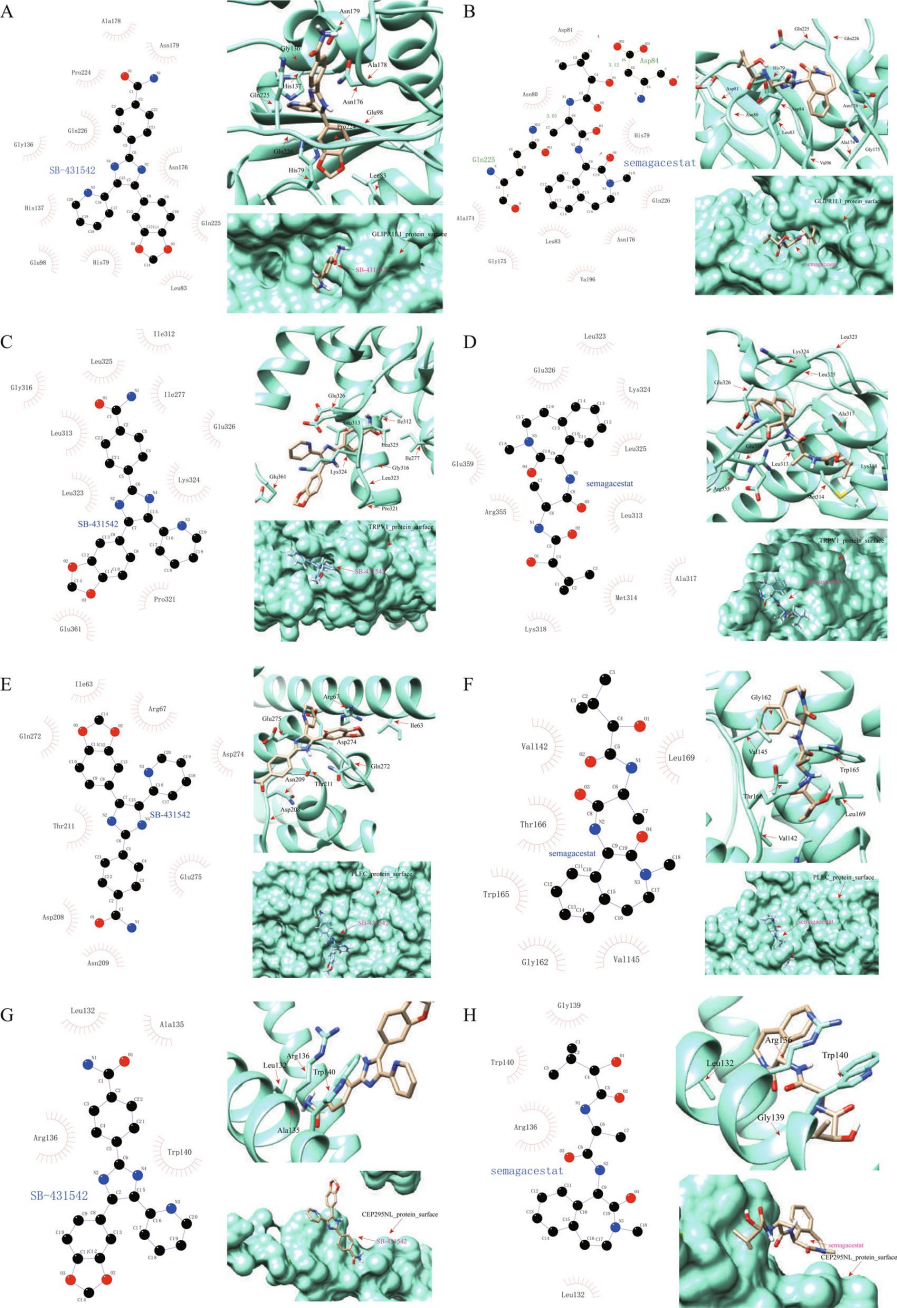
Binding of drug molecules with proteins encoded by ‘Downregulated in hot tumors, Prognostic, and Immune-Related Genes’. **A** GLIPR1L1 bound to SB-431542. **B** GLIPR1L1 bound to semagacestat. **C** TRPV1 bound to SB-431542. **D** TRPV1 bound to semagacestat. **E** PLEC bound to SB-431542. **F** PLEC bound to semagacestat. **G** CEP295NL bound to SB-431542. **H** CEP295NL bound to semagacestat

We subsequently downloaded the chemical structures of eight active compounds, namely, AT13387, carboplatin, CAY10594, thalidomide, TGX-221, dasatinib, semagacestat, and SB-431542, from the ZINC15 database. Then, we selected four genes with corresponding complete protein structures to explore the binding modes between the genes and drugs (Fig. 11A–D). Based on the docking score, the 2 drugs that exhibited the most potent binding with *GLIPR1L1* were SB-43154 and semagacestat (Supplementary Table 17). A pocket was identified on the surface of the GLIPR1L1 protein molecule, which allowed SB-43154 to interact with it to form a relatively stable complex (Fig. 11A), and another pocket on the surface of GLIPR1L1 interacted and formed a complex with semagacestat (Fig. 11B). According to 2D and 3D molecular interaction visualizations, semagacestat displayed more robust interactions with the Asp84 and Gln225 amino acids of GLIPR1L1 than did SB-43154. The 2 drugs with the most robust binding to TRPV1, PLEC, and CEP295NL were also SB-43154 and semagacestat (Supplementary Table 17). Although no pockets were identified on the surfaces of the TRPV1 and PLEC proteins, the binding of these proteins remained relatively stable due to the significant number of hydrogen bonds between the drugs and proteins (Fig. 11C–F). SB-43154 and semagacestat bound weakly to the CEP295NL protein, in which they interacted with only four amino acids (Fig. 11G, H, Supplementary Table 17).

### Validation of cell type-specific DPIRG expression in PAAD samples

**Fig. 12:**
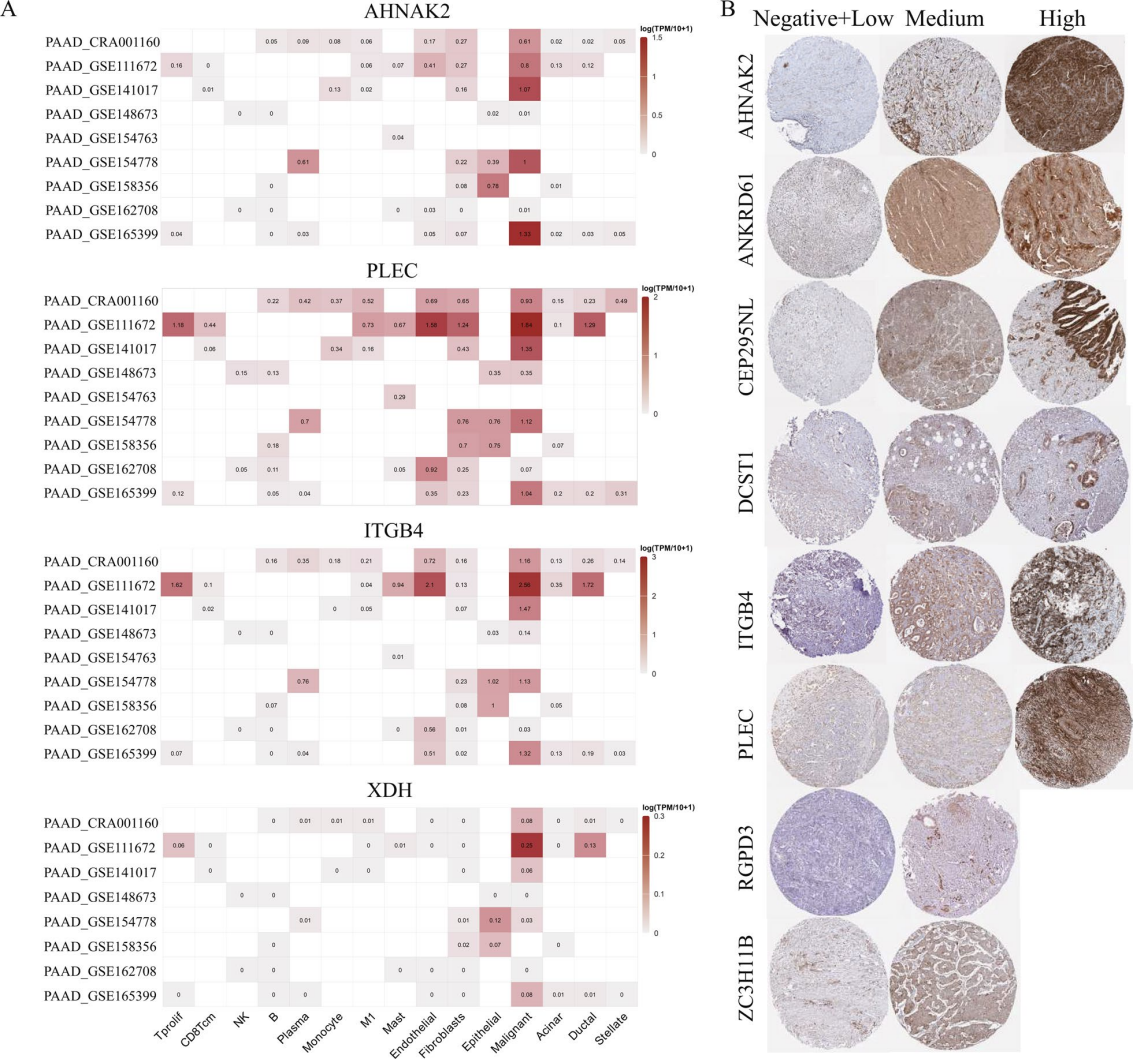
Single-cell RNA sequencing (scRNA-seq) and immunohistochemistry analyses. **A** RNA expression levels of *AHNAK2*, *PLEC*, *ITGB4*, and *XDH* in different cell types, based on data from nine scRNA-seq databases. **B** Protein expression levels of AHNAK2, ANKRD61, CEP295NL, DCST1, ITGB4, PLEC, RGPD3, and ZC3H11B in pancreatic adenocarcinoma tumor tissues

We downloaded eight PAAD scRNA-seq datasets, identified 15 cell types in the TME, and examined the cell type-specific expression of several DPIRGs that were differentially expressed between hot-low and cold-high tumors (Fig. 8C), particularly those whose expression was associated with genetic/epigenetic regulation (Fig. 6), including *AHNAK2*, *PLEC*, *ITGB4*, and *XDH.* We found that these genes were more strongly expressed in malignant cells (Fig. 12A). On the HPA website, IHC results were available for proteins corresponding to nine DPIRGs, including AHNAK2, ANKRD61, CEP295NL, DCST1, ITGB4, PLEC, RGPD3, and ZC3H11B. The distribution and staining intensity of these proteins in representative PAAD samples according to IHC analysis, ranging from low to high expression, are presented in Fig. 12B.

## Discussion

Over the last two decades, the incidence of PAAD has been increasing; however, the 5-year survival rate has not significantly improved [47]. There is accumulating evidence suggesting that ICB treatment efficacy is better in solid tumors with an inflamed TME than in those lacking immune infiltration [48]. Tumors with an inflamed TME featuring high immune cell infiltration are known as “hot” tumors, whereas “immune cell desert” or "immune cell excluded" tumors are commonly referred to as "cold" tumors. The lack of reliable biomarkers to differentiate between cold and hot tumors for personalized treatment has been a critical issue for clinicians [49]. The present study commenced by dividing patients with PAAD into two subgroups, immunologically hot and cold, based on their tumor immune compositions, with hot tumors having greater immune infiltration and being associated with better survival. We identified several genes that were differentially expressed between the two groups and were actively involved in tumor immunity. Based on these genes, we constructed a novel DPIRG signature, using which we developed a model that faithfully predicted the prognosis of patients with PAAD, suggesting that genes in the DPIRG signature have critical roles in tumor immunity and disease outcomes. Several PAAD prognostic signatures have been reported previously; however, most of these signatures involved genes regulating a few biological processes, such as 5-methylcytosine (m5C) modification, N6-methalogenorphine (M6A) regulation, and metabolic reprogramming [50–52], and did not consider the distinct immunological traits of hot and cold tumors or their impact on disease outcome.

ML can facilitate prognostic model establishment and help identify critical biomarkers [53]. In the present study, we established an ML framework and comprehensively analyzed the DPIRG signature in multiple independent PAAD cohorts, thereby establishing a robust and reliable prognostic model for patients with PAAD. By incorporating multiple ML algorithms, we also identified several biomarker genes that can distinguish hot tumors from cold tumors and predict PAAD prognosis, including *ITGB4*, *PLEC*, *TRPV1*, *AHNAK2*, *CEP295NL*, and *POLQ* (Fig. 7A–C).

*ITGB4* encodes the integrin beta4 protein, which is a receptor for laminin-5 that is expressed by a wide variety of cell types to facilitate G protein-coupled receptor binding and signal transduction [54]. Overexpression of *ITGB4* is correlated with poor prognosis in several cancers, including small-cell lung carcinoma [55], low-grade glioma [56], and hepatocellular carcinoma [57]. Furthermore, ITGB4 overexpression promotes epithelial-mesenchymal transition in pancreatic ductal adenocarcinoma [58]. Importantly, although the *ITGB4* gene mutation rate was only 2.5% in PAAD, CNV and DNA methylation of the *ITGB4* gene were positively and negatively correlated with *ITGB4* gene expression, respectively, and both were significantly associated with the survival of patients with PAAD, suggesting that both genetic and epigenetic mechanisms determine *ITGB4* levels and significantly impact disease outcome. Our findings support *ITGB4* as a biomarker for predicting the immune subtypes and prognosis of patients with PAAD; whether *ITGB4* contributes to the establishment of a cold immune microenvironment and whether targeting *ITGB4* could convert cold tumors to hot tumors in PAAD warrants further investigation.

Among the DPIRGs, *PLEC* had the highest mutation frequency in PAAD, at 9%*. PLEC* encodes the 500 kDa protein plectin, which has a multifunctional role in cellular organization and signal transduction [59]. Plectin has been reported to be a biomarker that is frequently overexpressed in some cancer types, including PAAD, lung cancer, and head and neck cancers [59]. In addition to its increased mutation frequency, we found that CNVs (predominantly amplification) of *PLEC* in PAAD samples were positively correlated with its expression levels. Further analysis of the scRNA-seq and protein databases confirmed that plectin is highly and predominantly expressed by cancer cells in PAAD specimens. Multiple studies have suggested that plectin exhibits protumorigenic activities by influencing cancer cell proliferation, migration, and invasion [59]; however, the role of plectin in modulating the tumor immune microenvironment has not been investigated. Herein, we found that *PLEC* is a crucial prognostic gene and biomarker that can distinguish PAAD immune subtypes, suggesting that it may play a crucial role in tumor immunity.

We also identified *TRPV1*, encoding transient receptor potential cation channel subfamily V member 1, as a critical prognostic DPIRG whose expression is upregulated in cold PAAD tumors and closely associated with CNVs of this gene. TRPV1 is a nonselective cation channel that can be activated by different physical and chemical stimuli [60]; it is commonly upregulated in several cancers, including tongue squamous cell cancer [61], PAAD [62], breast cancer [63], and prostate cancer [64]. Ion channels function to significantly modulate various biological processes, including intracellular calcium (Ca^2+^) and potassium (K^+^) levels, which regulate cell proliferation, migration, invasion, and apoptosis [65–68]. Although the underlying mechanisms are yet to be investigated, our results suggest that TRPV1 significantly influences the immune landscape of PAAD. Recent studies showing that TRPV1 is involved in the cross-talk between cancer and immune cells in the TME [69] and that targeting TRPV1 can effectively increase tumor immune infiltration by suppressing TGF-β signaling in pancreatic and breast cancer models [70] support this hypothesis. Overall, the biomarkers identified in the present study provide a rich resource for future studies to improve PAAD stratification according to immune subtype and targeted therapy.

Although some miRNAs have been found to have roles in pancreatic cancer cell proliferation, invasion, and metastasis, there has been limited research on their ability to modulate hot/cold tumor immune microenvironments. Here, we identified several miRNAs that were positively correlated with DPIRG expression levels, among which hsa-mir-193a, hsa-mir-1248, hsa-mir-365a, hsa-mir-365b, and hsa-mir-93 were expressed at higher levels in the cold-high group, while hsa-mir-139 was more strongly expressed in the hot-low group. MiRNAs can have anti- or protumorigenic properties in different contexts. For example, hsa-mir-365a was reported to promote lung tumorigenesis, and inhibition of hsa-miR-365a suppressed lung cancer cell proliferation, migration, and invasion [71]. In addition, M2 macrophages in the TME secrete miR-365 through extracellular vesicles, thus promoting pancreatic ductal adenocarcinoma progression through activation of the BTG2/FAK/AKT axis [72]. Hsa-mir-93 upregulation can promote pancreatic cancer cell proliferation and invasion while inducing resistance to chemotherapy by directly inhibiting PTEN [73].

In contrast, hsa-mir-139 has been identified as a tumor suppressor in several cancers, including acute myeloid leukemia, liver cancer, and lung cancer [74]. Furthermore, hsa-miR-139-5p was reported to suppress the epithelial-mesenchymal transition and metastasis of pancreatic cancer cells [75]. More experiments are needed to clarify whether these miRNAs are downstream targets of DPIRGs and whether they are responsible for DPIRG-mediated immunosuppression in PAAD.

Finally, our analysis centered on ML algorithms to pinpoint the top potential drugs that could significantly impact disease prognosis or DPIRG expression. Four drugs, namely, thalidomide, SB-431542, bleomycin A2, and ML258, overlapped in terms of prognosis and DPIRG score analysis, suggesting that they could have therapeutic effects on PAAD through modulating the TME. Thalidomide reportedly promotes tumor progression in PAAD by inhibiting epithelial-to-mesenchymal transition [76]. SB-431542 targets the TGF-β pathway, which is involved in cancer cell proliferation, differentiation, and apoptosis, as well as extracellular matrix constitution in the TME [77]. Bleomycin belongs to a family of antineoplastic antibiotics used to treat various types of cancer and can cause DNA breaks that can lead to cell death [78]. Although PAAD is not typically treated with bleomycin A2 [78], this drug may have beneficial effects by modulating the TME through the induction of immunogenic cell death [79]. In future research, investigations into the use of these drugs to treat PAAD or to transform cold tumors into hot tumors in PAAD are needed. In addition, understanding the opposing and synchronized effects of these drugs will provide evidence to inform appropriate medical interventions for treating PAAD [80].

## Conclusions

Immune cell deconvolution, consensus clustering, and immune profiling were employed to classify patients with PAAD into groups with immunologically hot and cold tumors who had distinct disease outcomes. By examining differentially expressed, immune-related, and prognostic genes between hot and cold tumors, we identified a novel DPIRG gene signature, which was successfully employed to generate a consensus ML framework for the prediction of prognosis in patients with PAAD. Moreover, ML was used to identify critical TME-regulating biomarker genes, and their molecular mechanisms were explored by integrative analysis, thereby providing new insights into the complex network of tumor-immune interactions occurring in the PAAD TME. In addition, we conducted drug sensitivity and molecular docking studies to identify drug candidates and corresponding protein targets in PAAD. These drugs have the potential for future application in the treatment of PAAD through the transformation of immunologically cold tumors into hot tumors.

### Supplementary Information

Additional file 1: Fig. S1. Consensus clustering analysis. **A** The consistency cumulative distribution functionplot of different k value. **B** Delta Area Plot displays the relative change of the area under the CDF curve compared to k and k-1. **C** The cluster consensus plot shows the cluster-consensus value of each cluster under different k values.

Additional file 2: Fig. S2. Differences in the fractions of immune cells between the two clusters

Additional file 3: Fig. S3. HR and p values of **A** UPIRGs and **B** DPIRGs in the TCGA and four GSE datasets.

Additional file 4: Fig. S4. The risk score and survival status of high- and low-risk PAAD patients in the four GSE datasets. **A**, **B** GSE28735, **C**, **D** GSE62452, **E**, **F** GSE78229, and **G**, **H** GSE85916.

Additional file 5: Fig. S5. Results of five machine learning methods based on mixed PAAD patients. **A** RF. **B**, **C** Boruta. **D** ANN. **E** SVM. **F** XGboost.

Additional file 6: Fig. S6. Results of five machine learning methods based on hot-tumor PAAD patients. **A** RF. **B**, **C** Boruta. **D** ANN. **E** SVM. **F** XGboost.

Additional file 7: Fig. S7. Results of five machine learning methods based on cold-tumor PAAD patients. **A** RF. **B**, **C** Boruta. **D** ANN. **E** SVM. **F** XGboost.

Additional file 8: Fig. S8. ROC curves to predict hot and cold tumor conditions with DPIRGs

Additional file 9: Fig. S9. ROC curves to predict hot-low and cold-high tumor conditions with DPIRGs

Additional file 10: Fig. S10. Correlations between DPIRGs and three immune scores in the hot-low and cold-high groups

Additional file 11: Fig. S11. The correlations between RNA expression levels of DPIRGs and predicted drug responses

Additional file 12: Table S1. Up differentially expressed genes between hot tumor and cold tumor of PAAD patients

Additional file 13: Table S2. Down differentially expressed genes between hot tumor and cold tumor of PAAD patients

Additional file 14: Table S3. Correlation coefficients and *P*-values between DPIRGs in hot-low group.

Additional file 15: Table S4. Correlation coefficients and *P*-values between DPIRGs in cold-high group.

Additional file 16: Table S5. Correlation coefficients and *P*-values between DPIRGs and DPIRGs-score in hot-low group.

Additional file 17: Table S6. Correlation coefficients and *P*-values between DPIRGs and DPIRGs-score in cold-high group.

Additional file 18: Table S7. Correlation coefficients and *P*-values between DPIRGs and risk score in hot-low group.

Additional file 19: Table S8. Correlation coefficients and *P*-values between DPIRGs and risk score in cold-high group.

Additional file 20: Table S9. Correlation coefficients and *P*-values between immune cells in hot-low group.

Additional file 21: Table S10. Correlation coefficients and *P*-values between immune cells in cold-high group.

Additional file 22: Table S11. Correlation coefficients and *P*-values between immune cells and DPIRGs-score in hot-low group.

Additional file 23: Table S12. Correlation coefficients and *P*-values between immune cells and DPIRGs-score in cold-high group.

Additional file 24: Table S13. Correlation coefficients and *P*-values between immune cells and risk score in hot-low group.

Additional file 25: Table S14. Correlation coefficients and *P*-values between immune cells and risk score in cold-high group.

Additional file 26: Table S15. Correlation coefficients between DPIRGs and predicted drugs of PAAD patients

Additional file 27: Table S16. The *P*-values between DPIRGs and predicted drugs of PAAD patients.

Additional file 28: Table S17. The docking scores between drugs and proteins

## Data Availability

The mRNA expression profile, mutation annotation, CNV, and clinical metadata data were obtained from TCGA-PAAD via the University of California-Santa Cruz (UCSC) browser (https://xenabrowser.net/). RNA-seq data have been deposited in ICGC (https://dcc.icgc.org/) and the Gene Expression Omnibus (GEO) with accession numbers GSE85916, GSE28735, GSE62452, and GSE78229. ScRNA-seq data have been deposited with the accession numbers CRA001160, GSE111672, GSE141017, GSE148673, GSE154778, GSE158356, GSE162708, and GSE165399. The full code used during the current study is available at https://github.com/sangmm12/ML_Hot-cold.
