# Supplementary figures and images for: Machine learning-based identification of biomarkers and drugs in immunologically cold and hot pancreatic adenocarcinomas
Source: J Transl Med. 2024 Aug 16;22:775. doi: 10.1186/s12967-024-05590-0 (PMC11328457; doi:10.1186/s12967-024-05590-0)

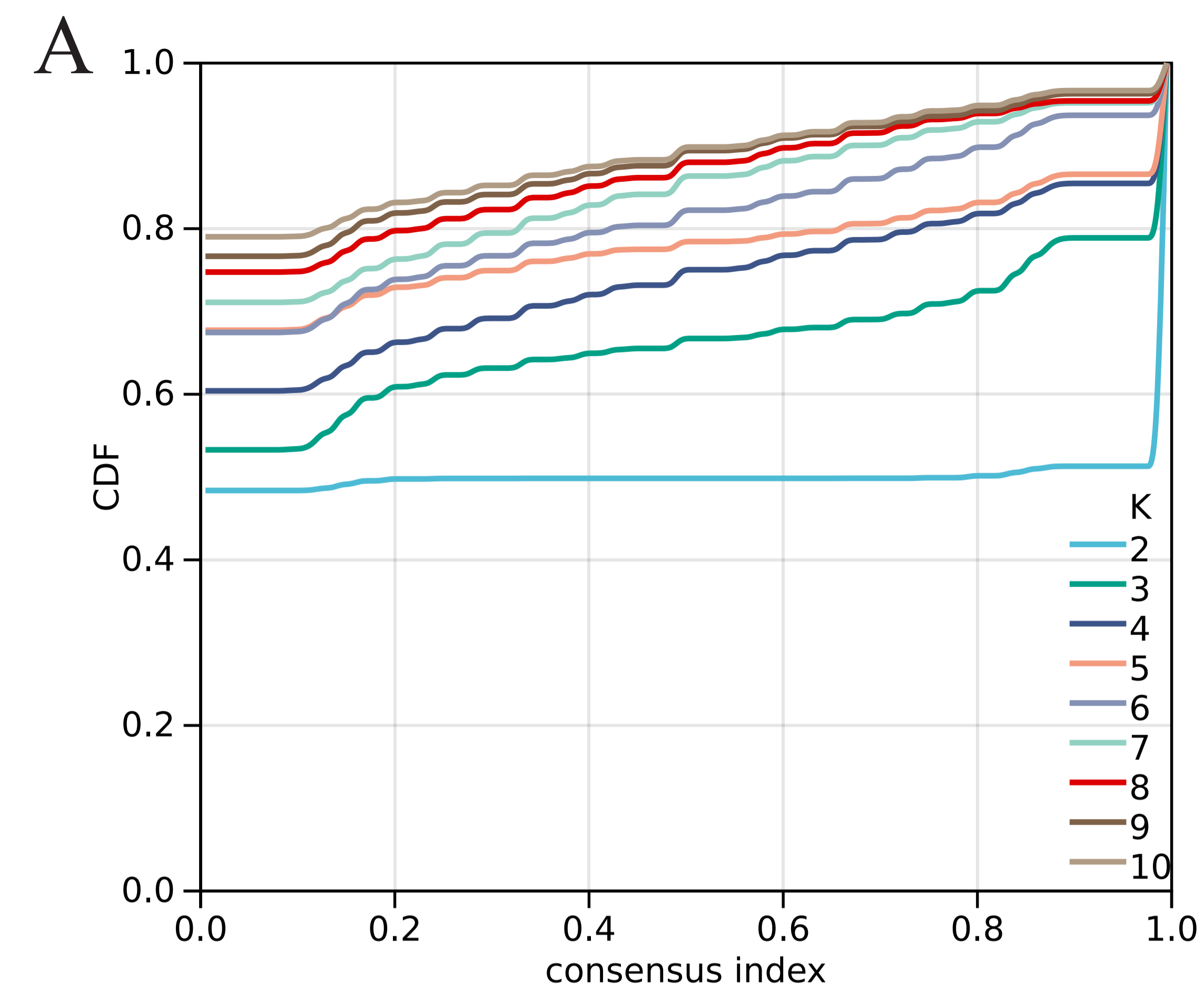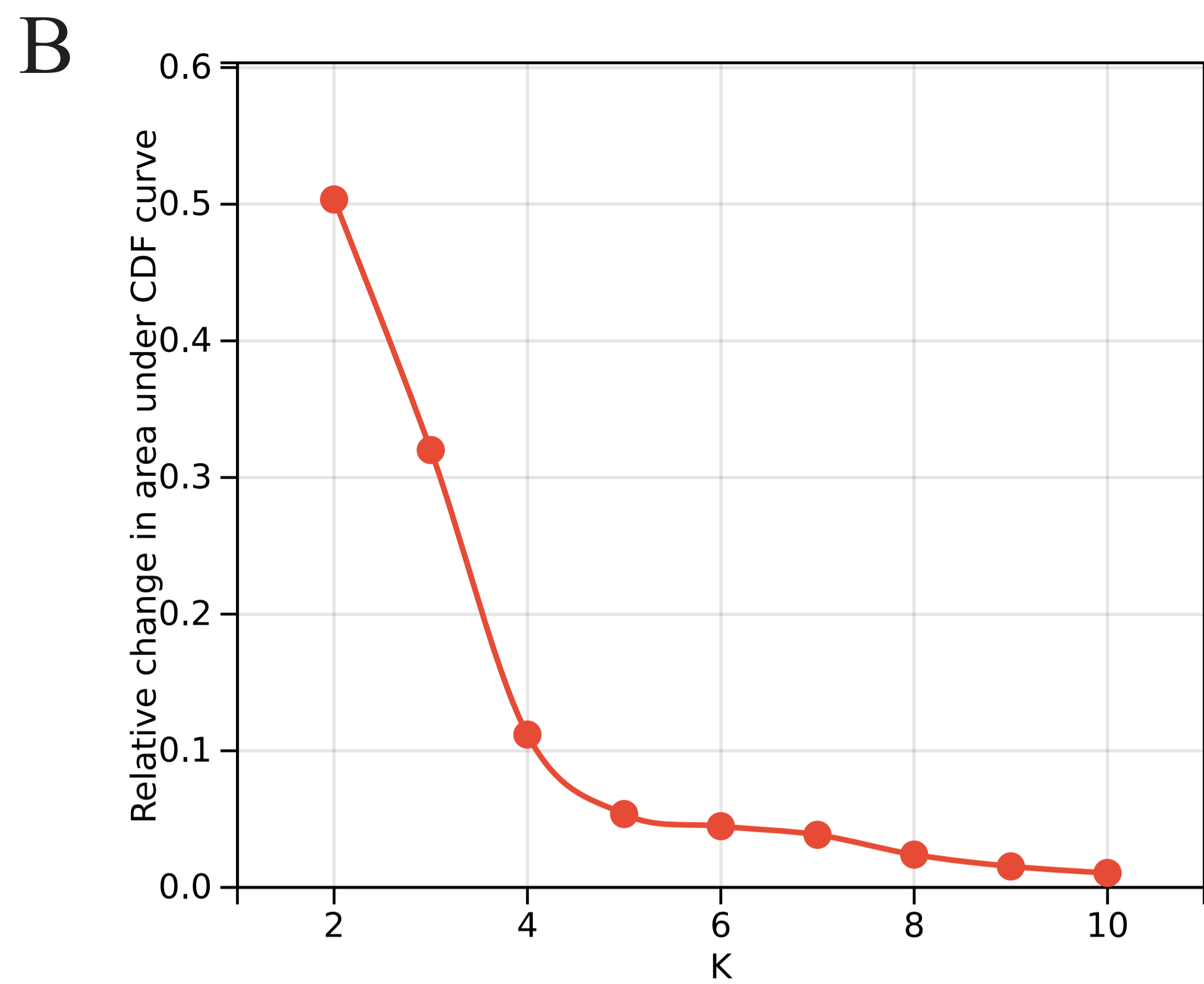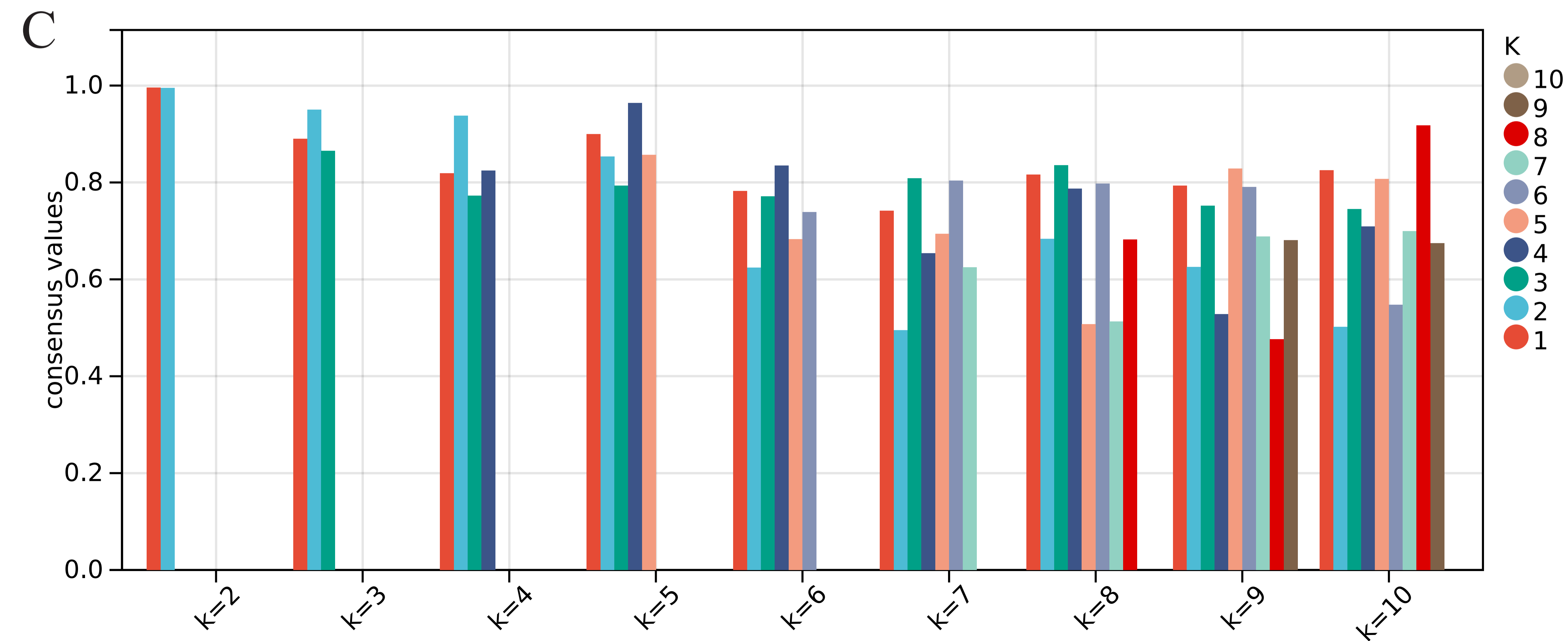

Supplement: Supplementary file 1 — Additional file 1: Fig. S1. Consensus clustering analysis. A The consistency cumulative distribution functionplot of different k value. B Delta Area Plot displays the relative change of the area under the CDF curve compared to k and k-1. C The cluster consensus plot shows the cluster-consensus value of each cluster under different k values. [file 12967_2024_5590_MOESM1_ESM.pdf]

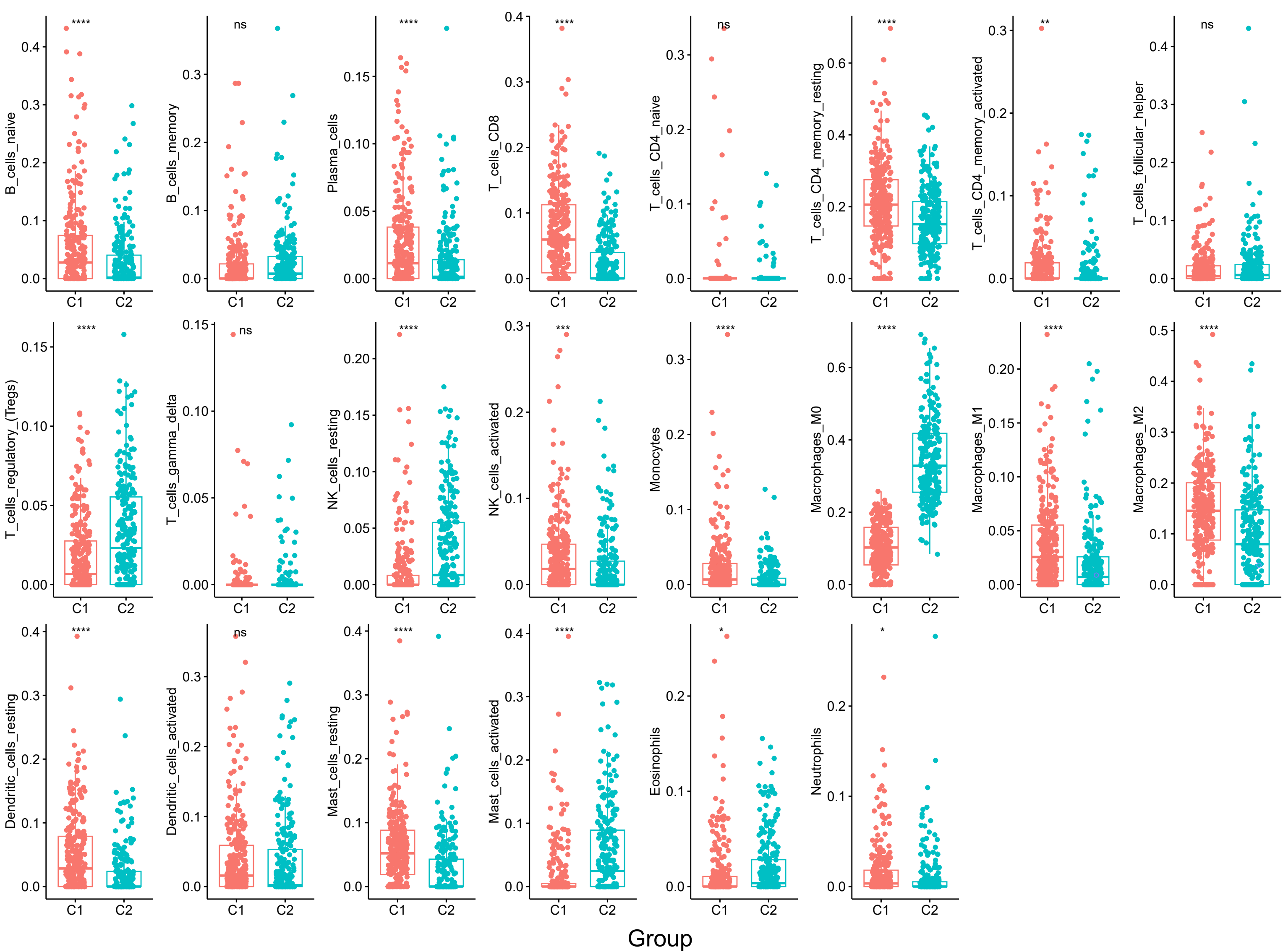

Supplement: Supplementary file 2 — Additional file 2: Fig. S2. Differences in the fractions of immune cells between the two clusters [file 12967_2024_5590_MOESM2_ESM.pdf]

A

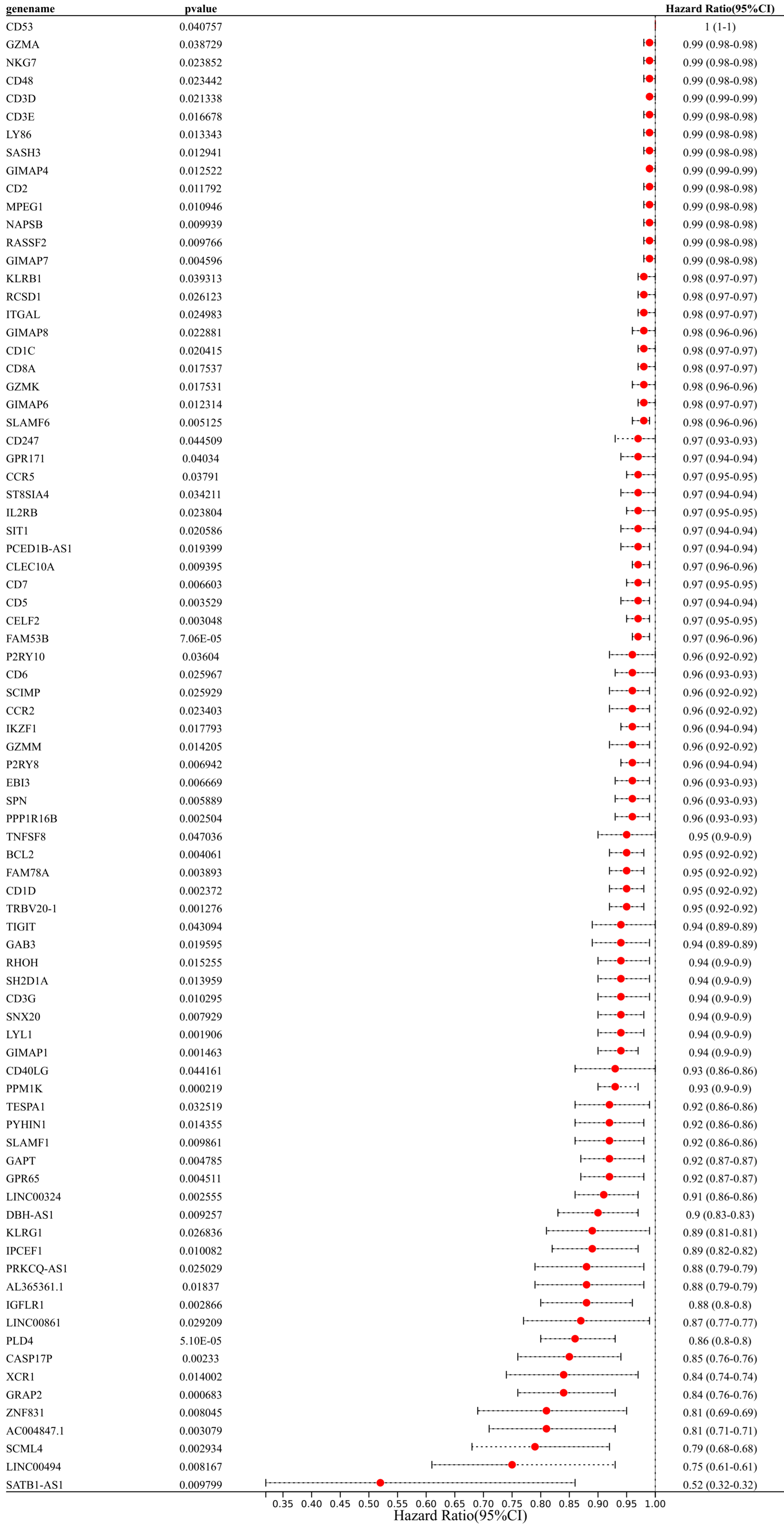

B

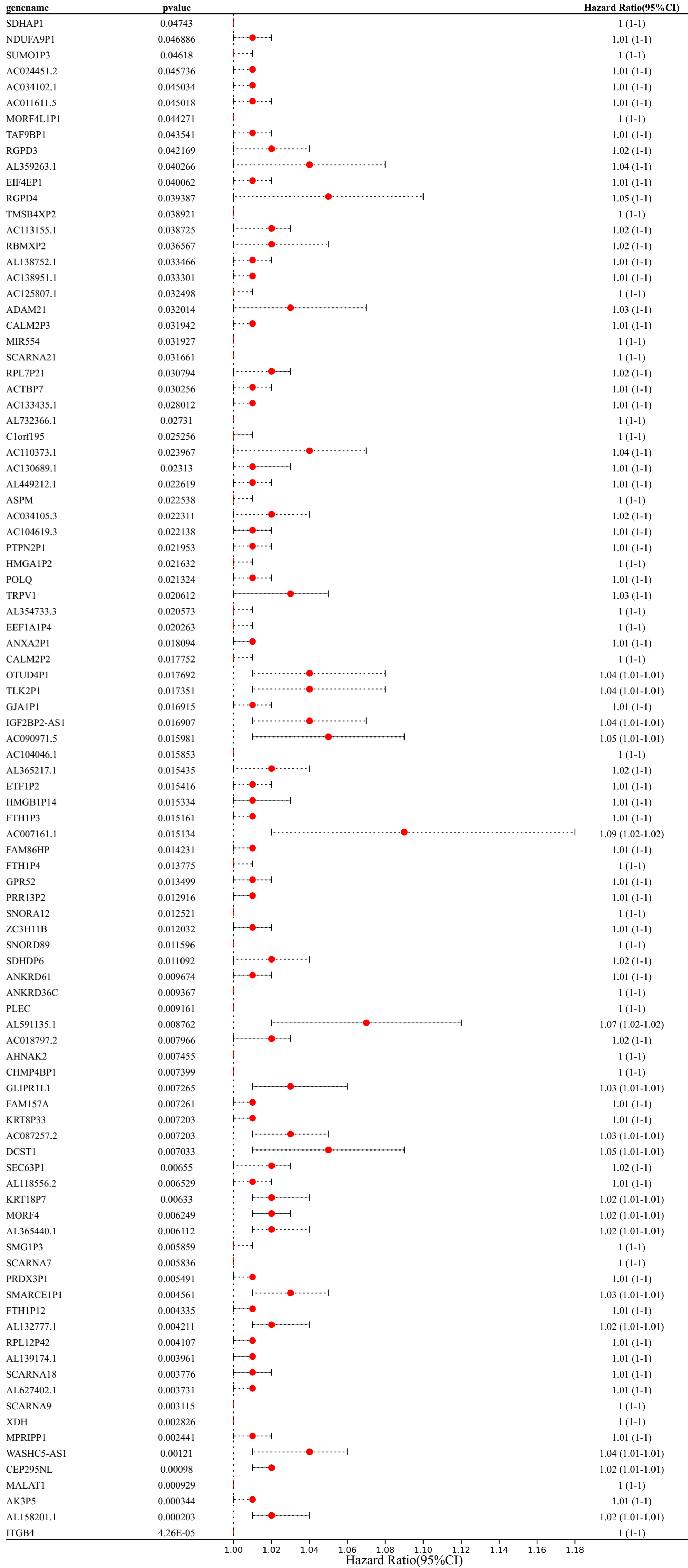

Supplement: Supplementary file 3 — Additional file 3: Fig. S3. HR and p values of A UPIRGs and B DPIRGs in the TCGA and four GSE datasets. [file 12967_2024_5590_MOESM3_ESM.pdf]

TCGA\_ICGC

A

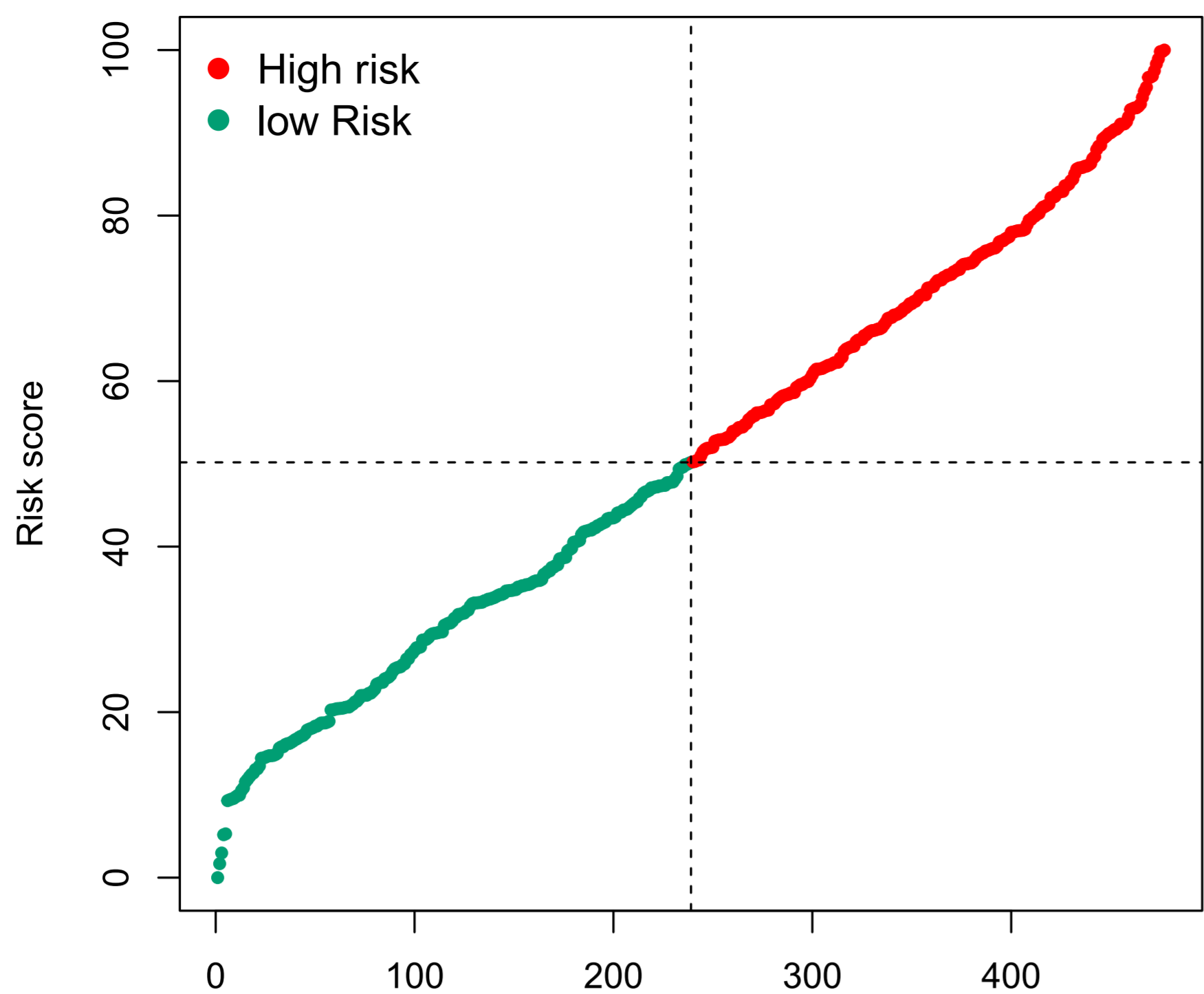

B

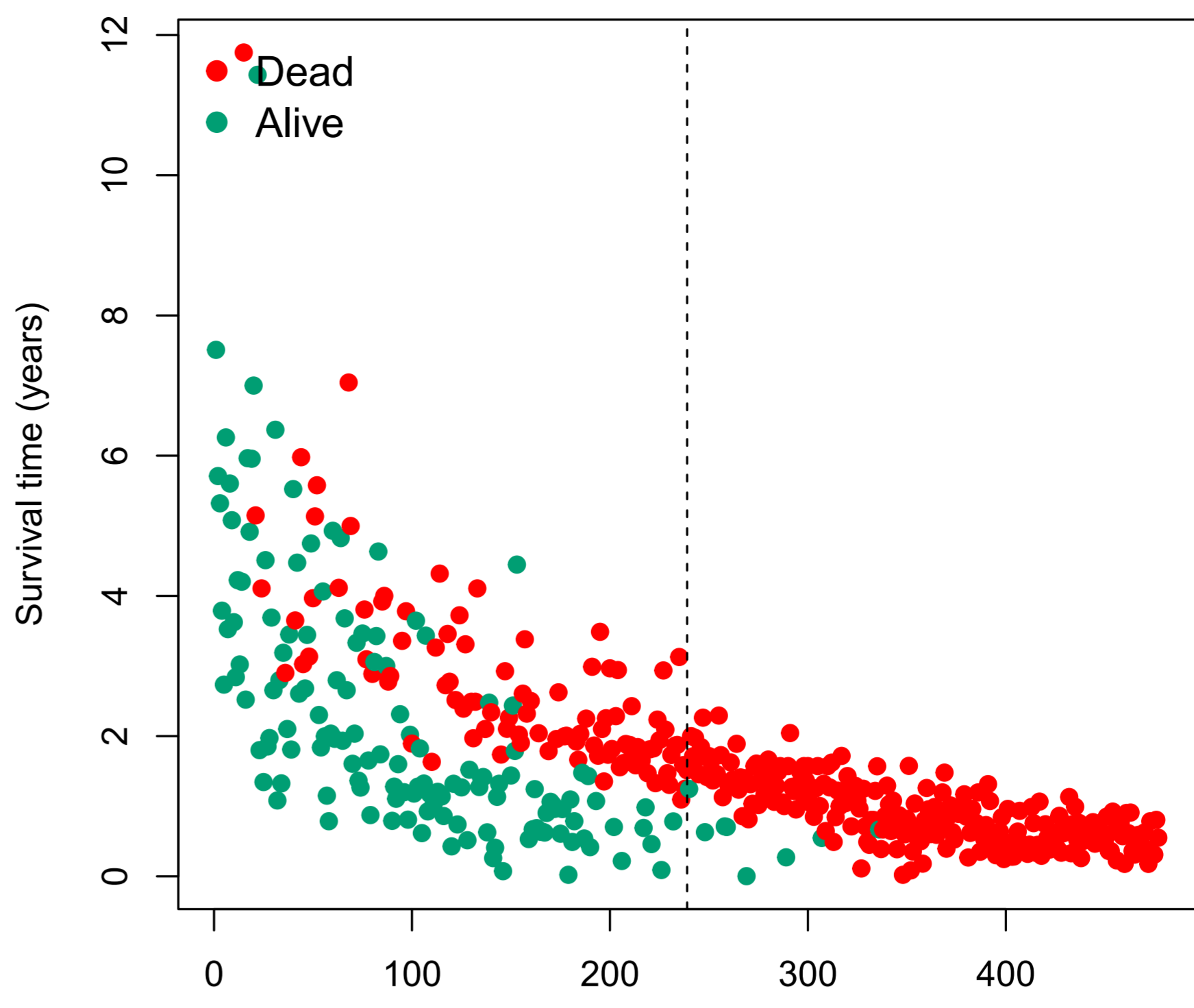

GSE28735

C

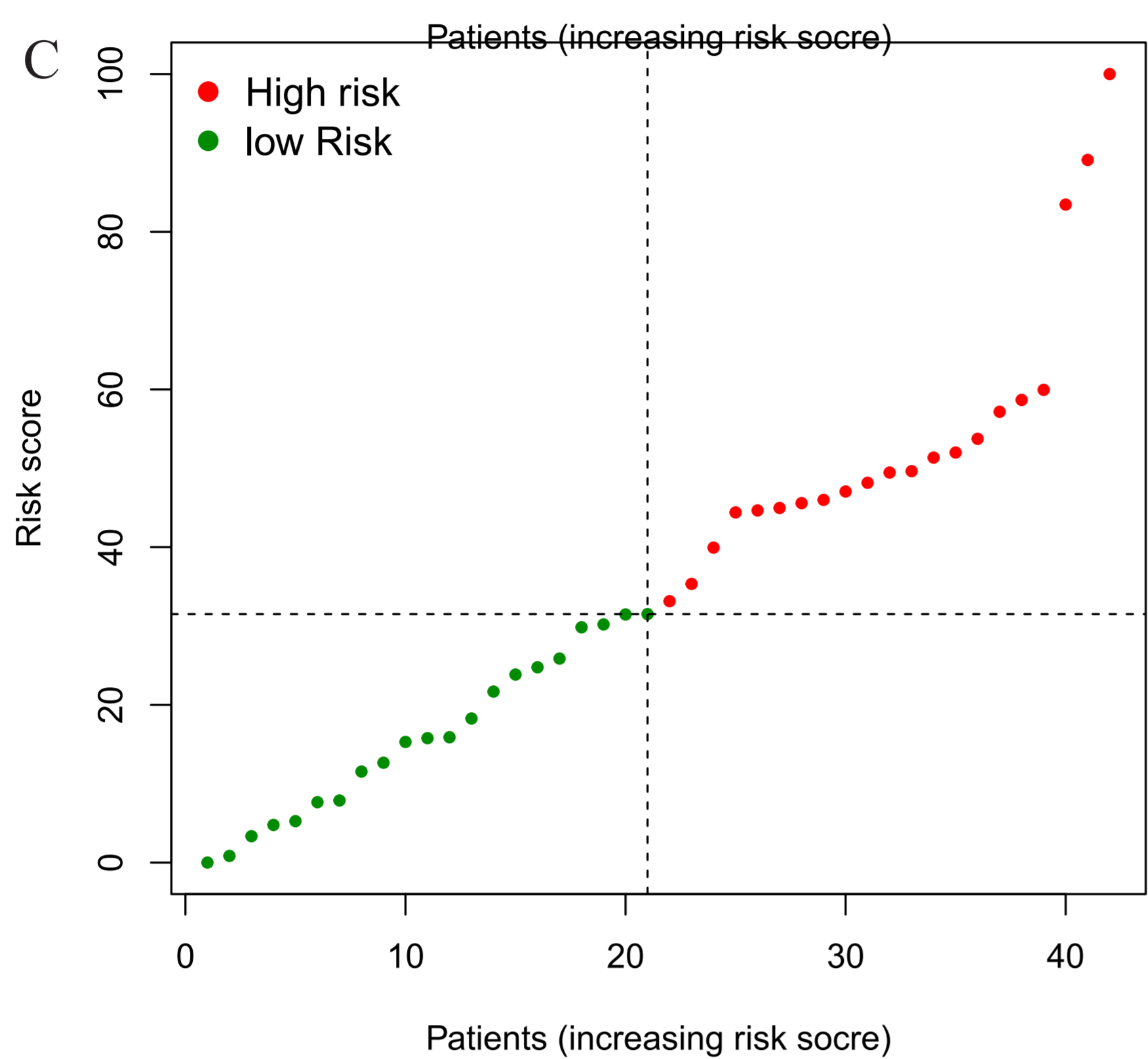

D

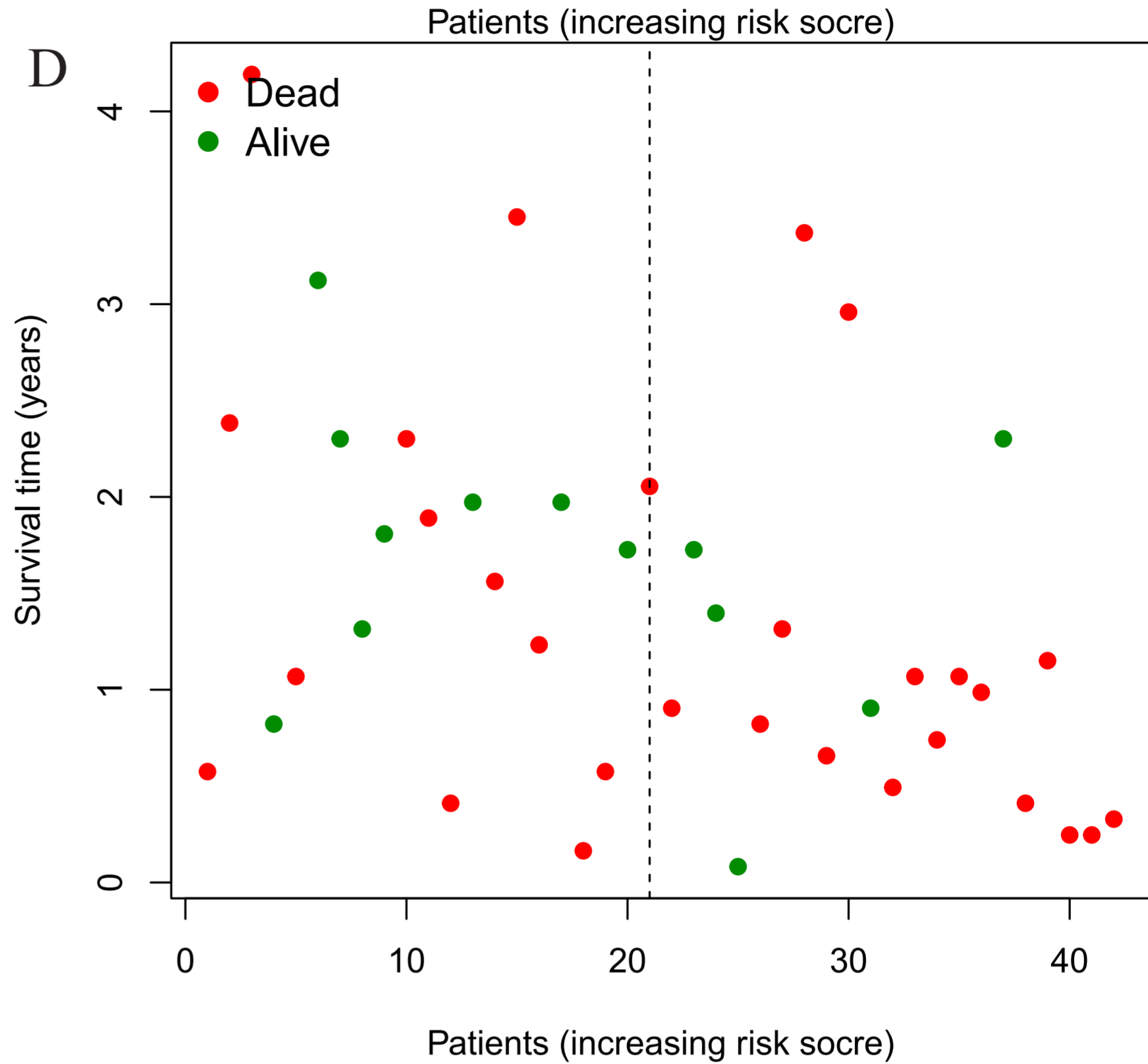

GSE62452

E

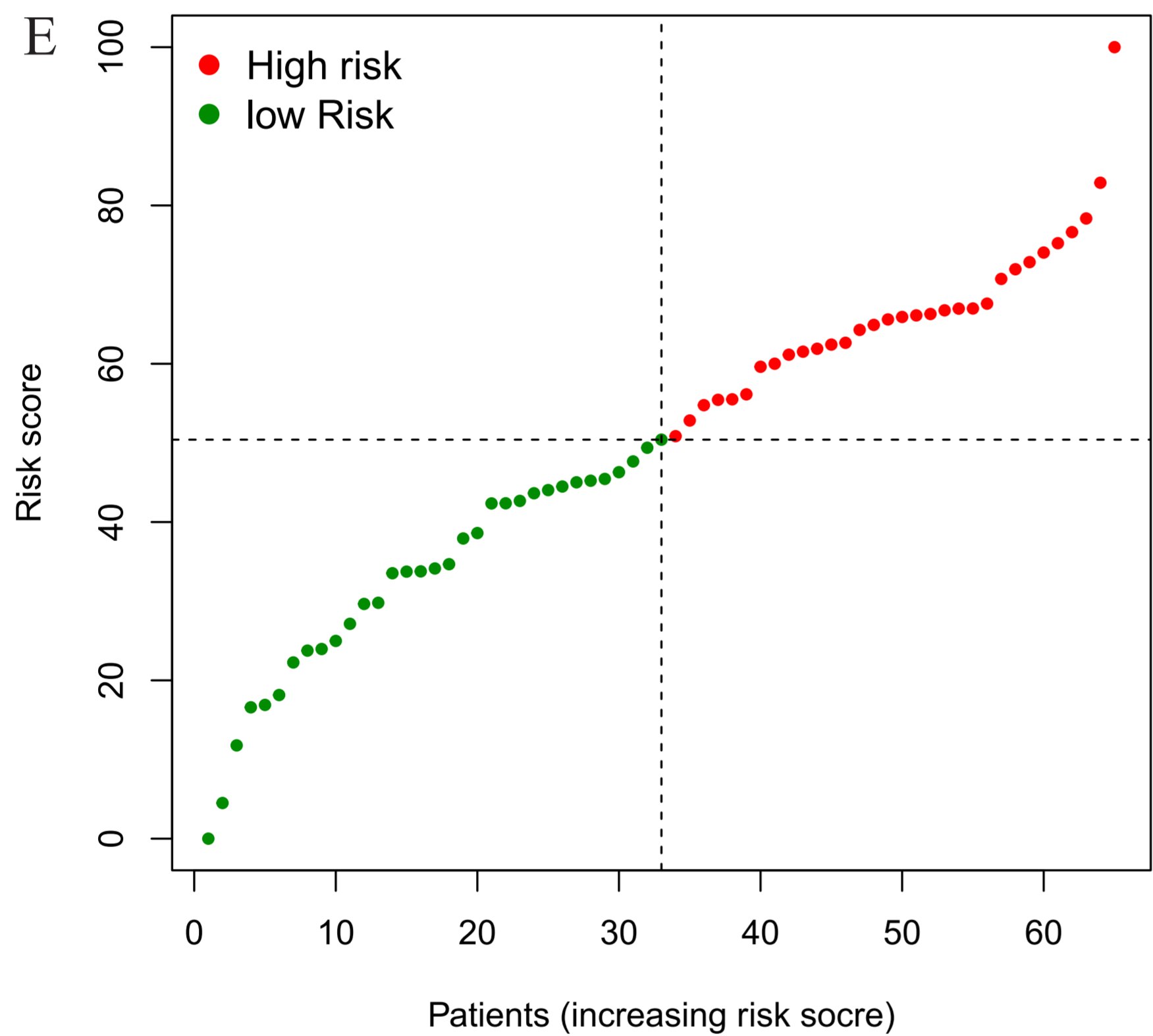

F

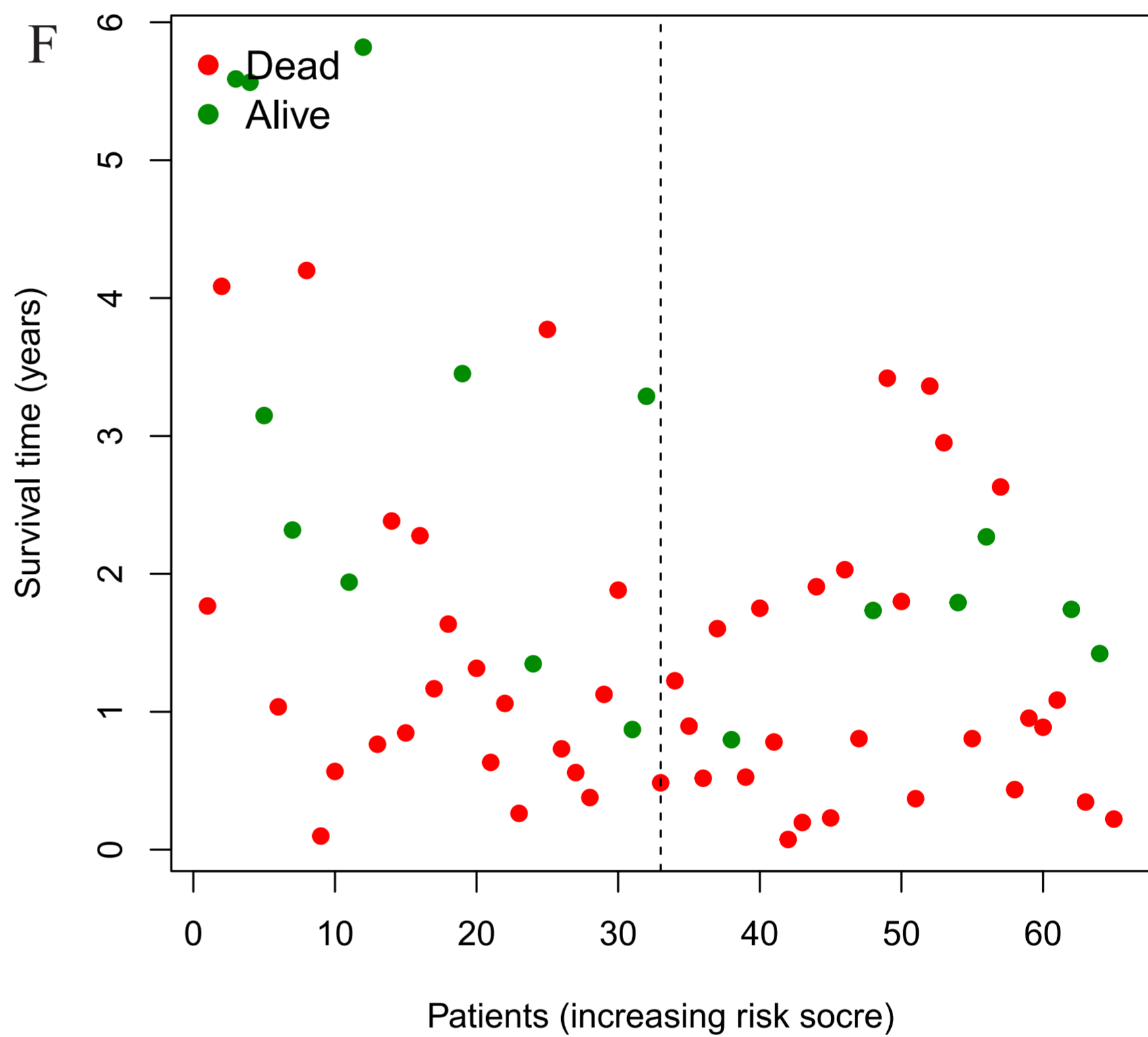

GSE78229

G

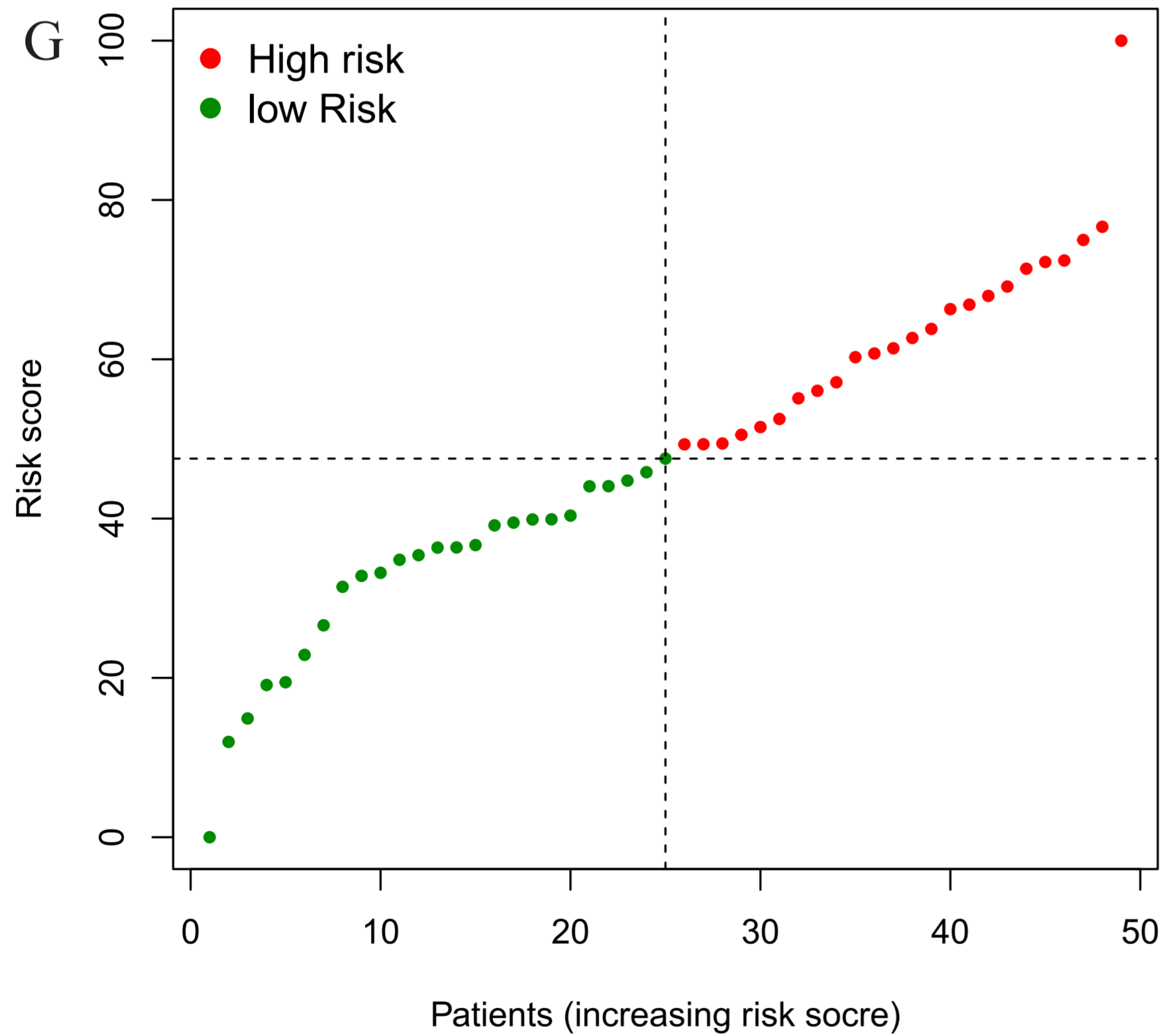

H

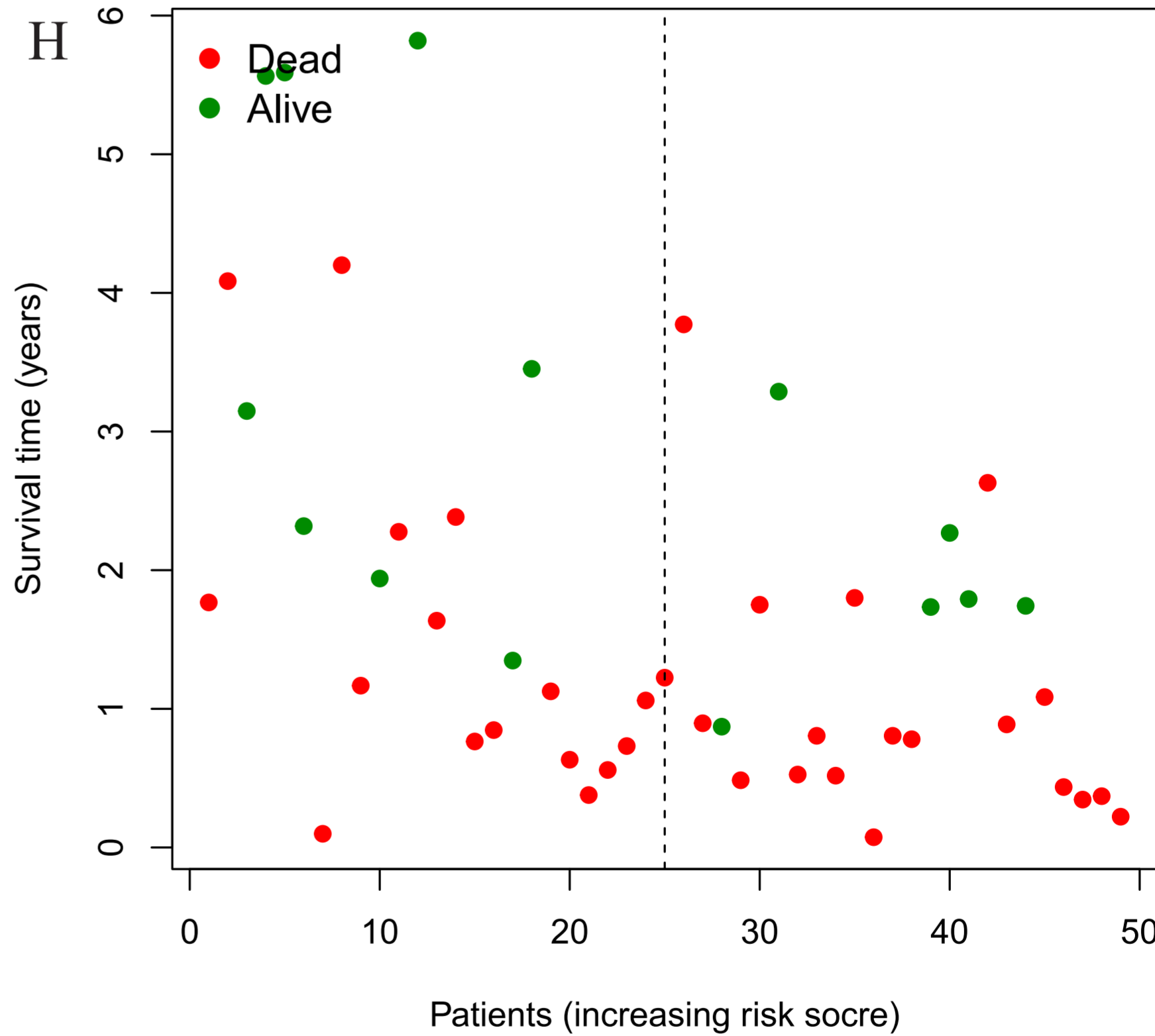

GSE85916

I

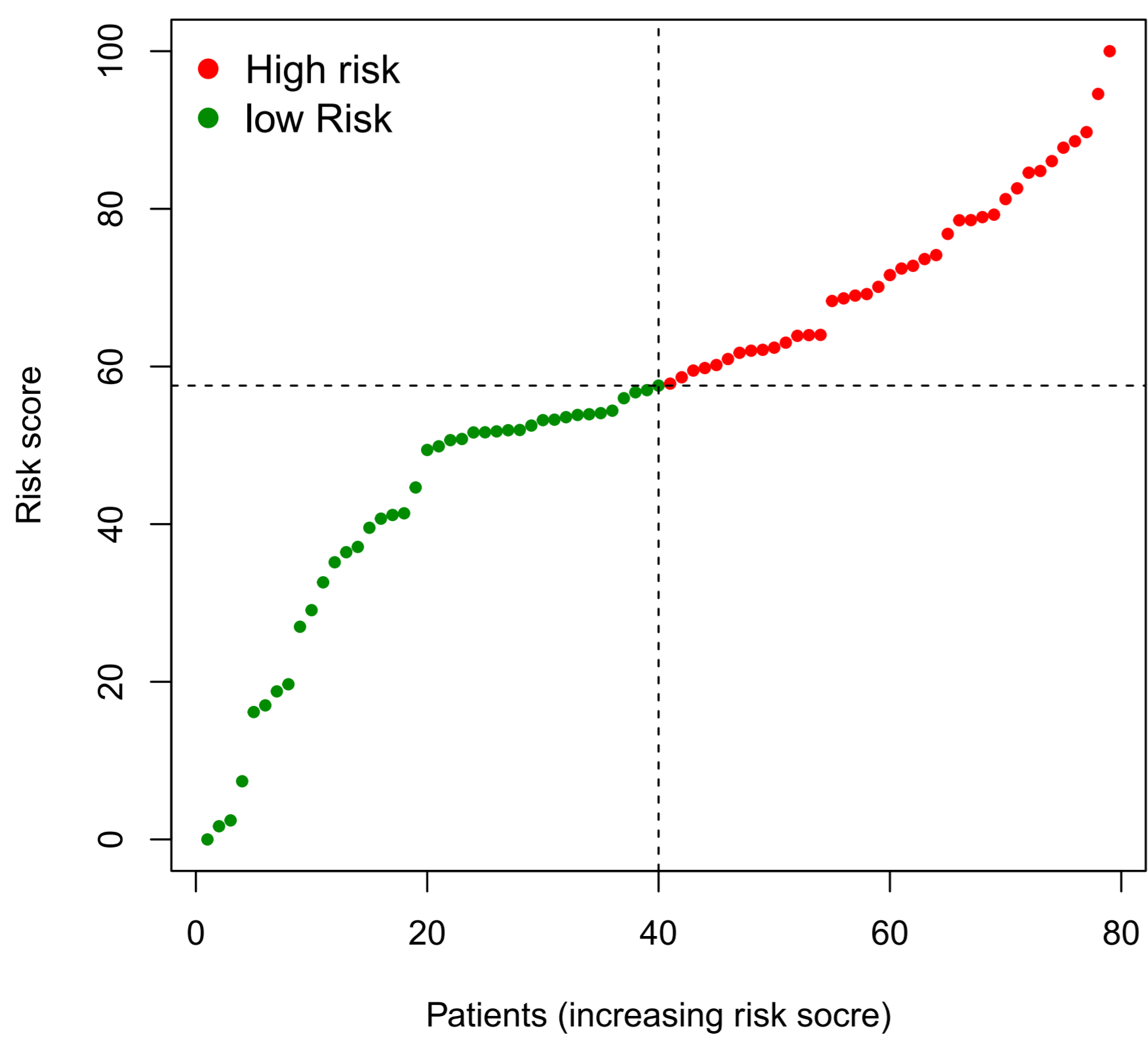

J

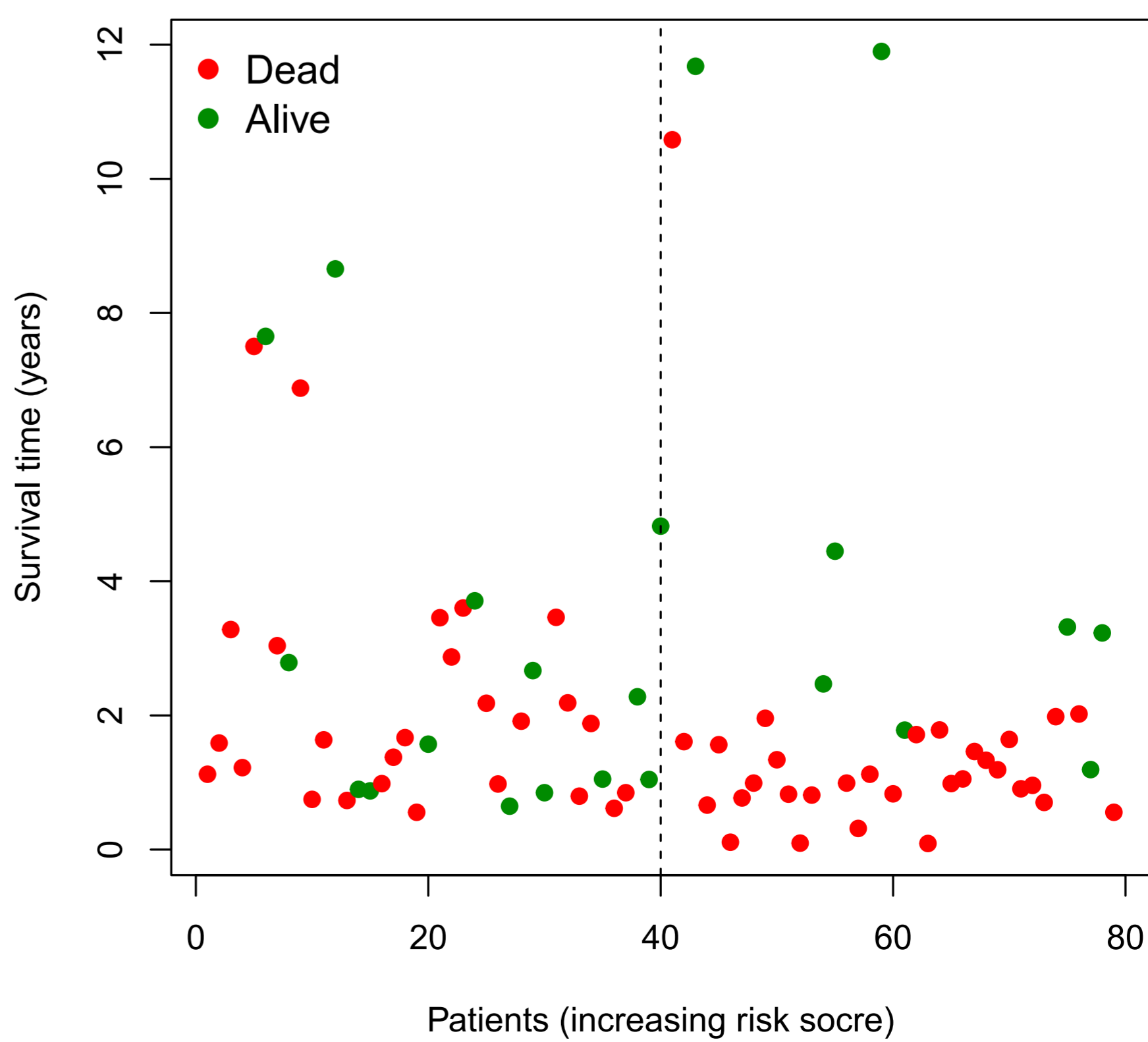

Supplement: Supplementary file 4 — Additional file 4: Fig. S4. The risk score and survival status of high- and low-risk PAAD patients in the four GSE datasets. A, B GSE28735, C, D GSE62452, E, F GSE78229, and G, H GSE85916. [file 12967_2024_5590_MOESM4_ESM.pdf]

A

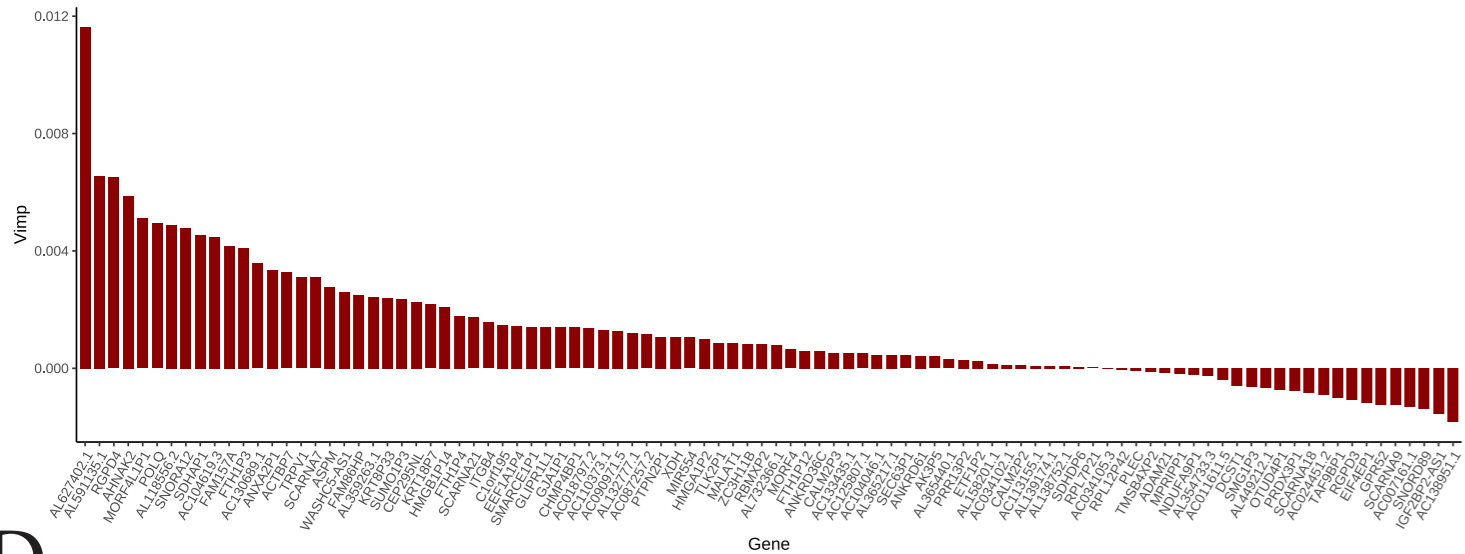

D

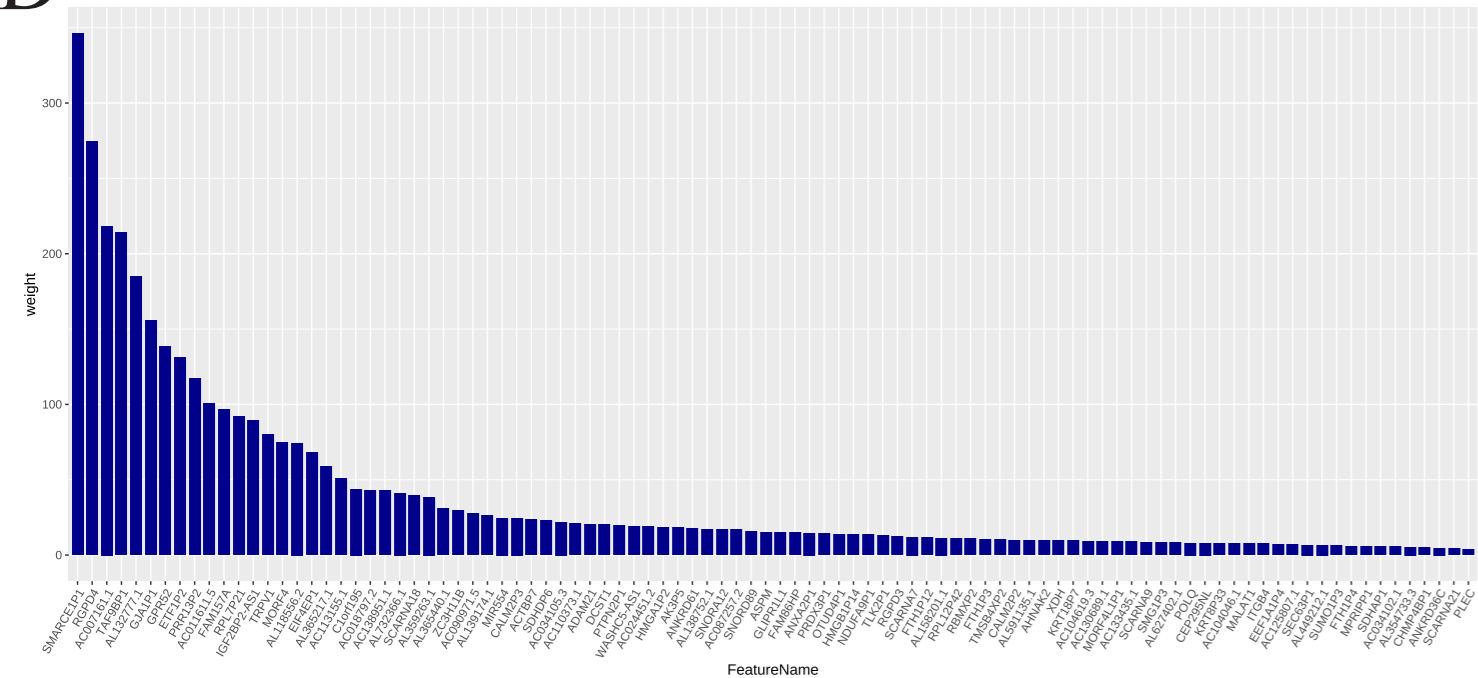

B

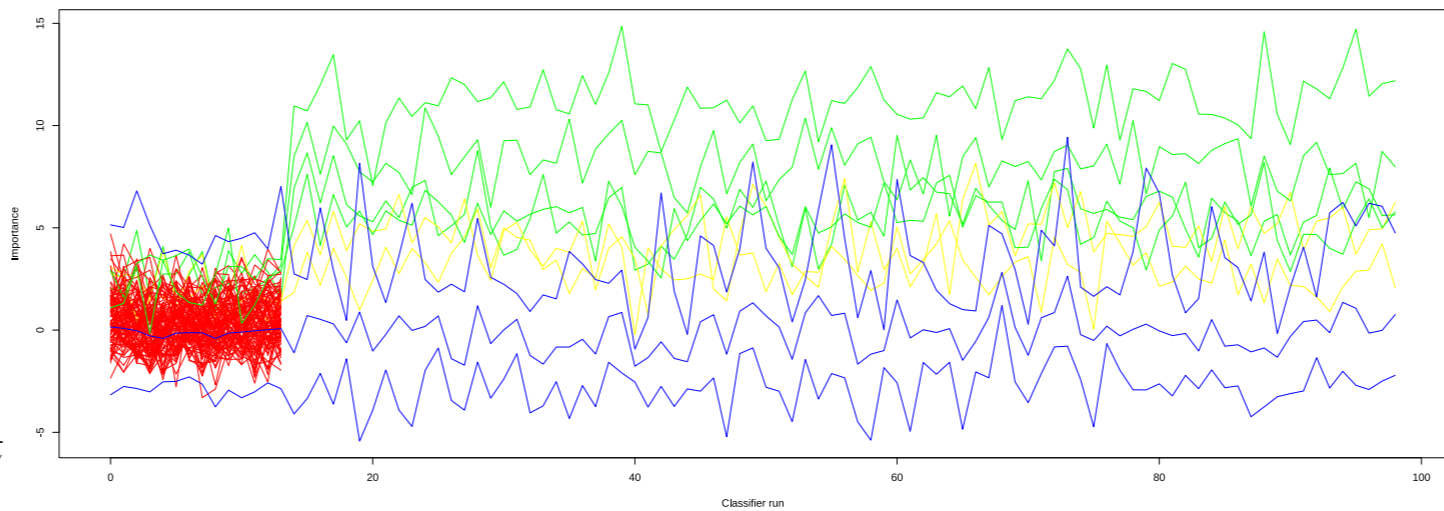

E

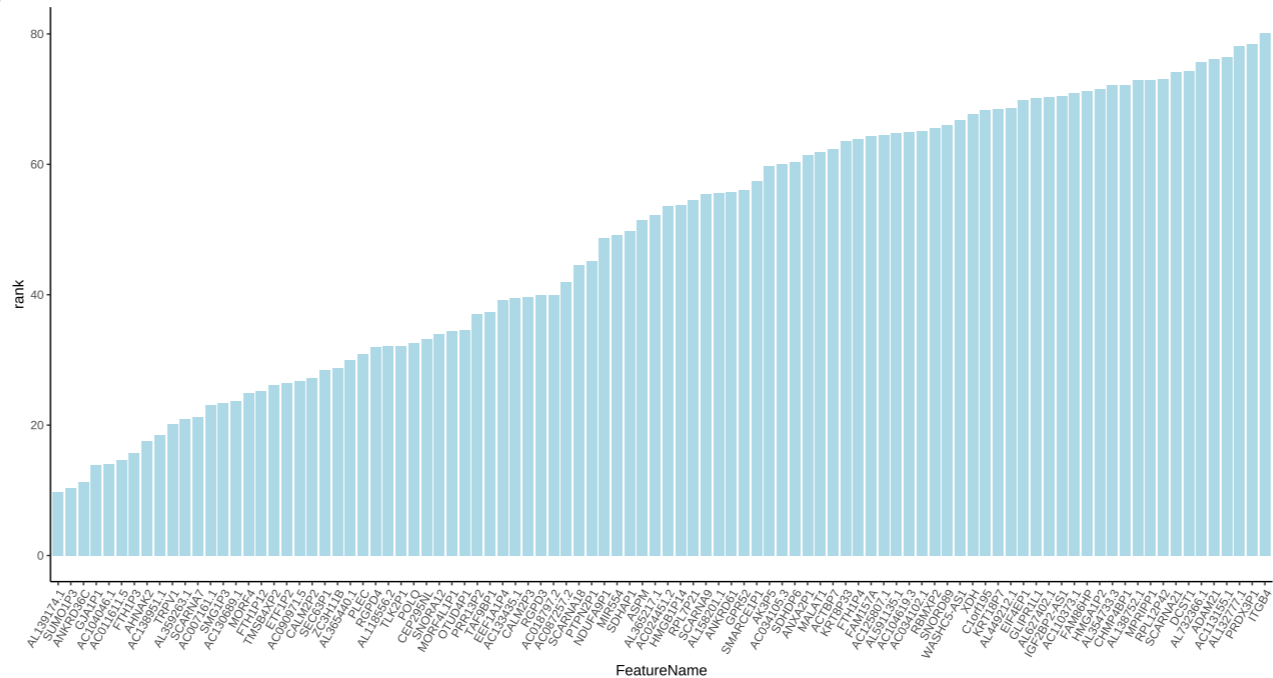

C

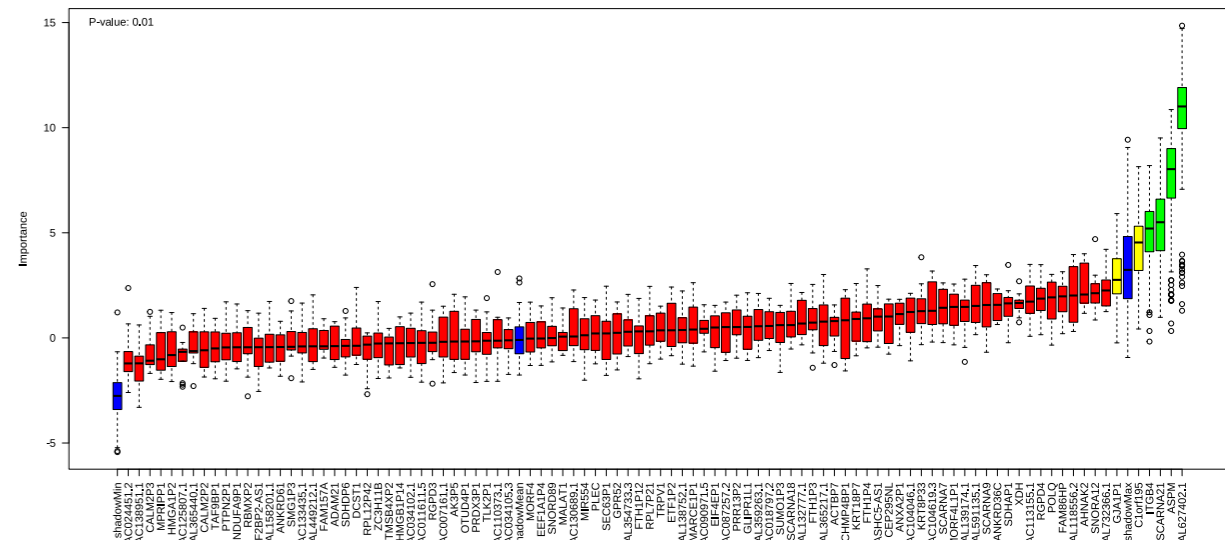

F

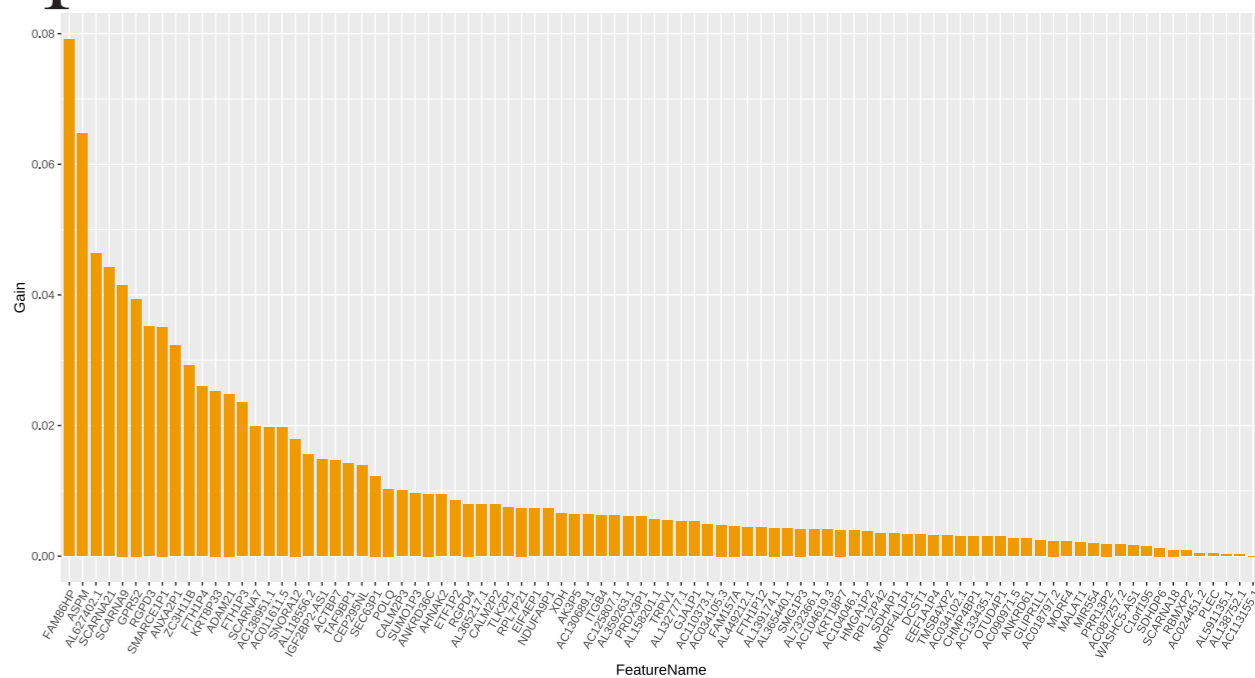

Supplement: Supplementary file 6 — Additional file 6: Fig. S6. Results of five machine learning methods based on hot-tumor PAAD patients. A RF. B, C Boruta. D ANN. E SVM. F XGboost. [file 12967_2024_5590_MOESM6_ESM.pdf]

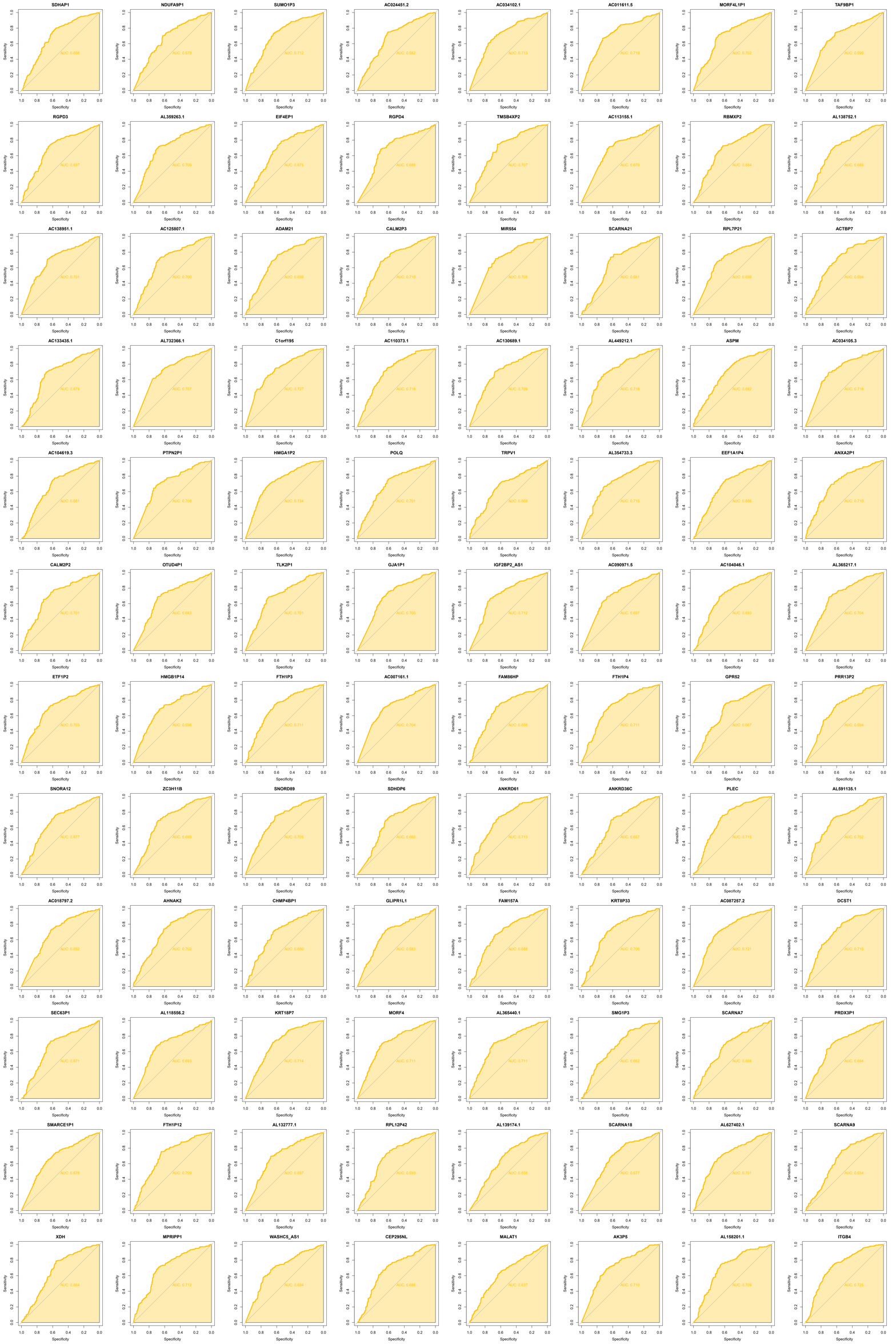

Supplement: Supplementary file 8 — Additional file 8: Fig. S8. ROC curves to predict hot and cold tumor conditions with DPIRGs [file 12967_2024_5590_MOESM8_ESM.pdf]

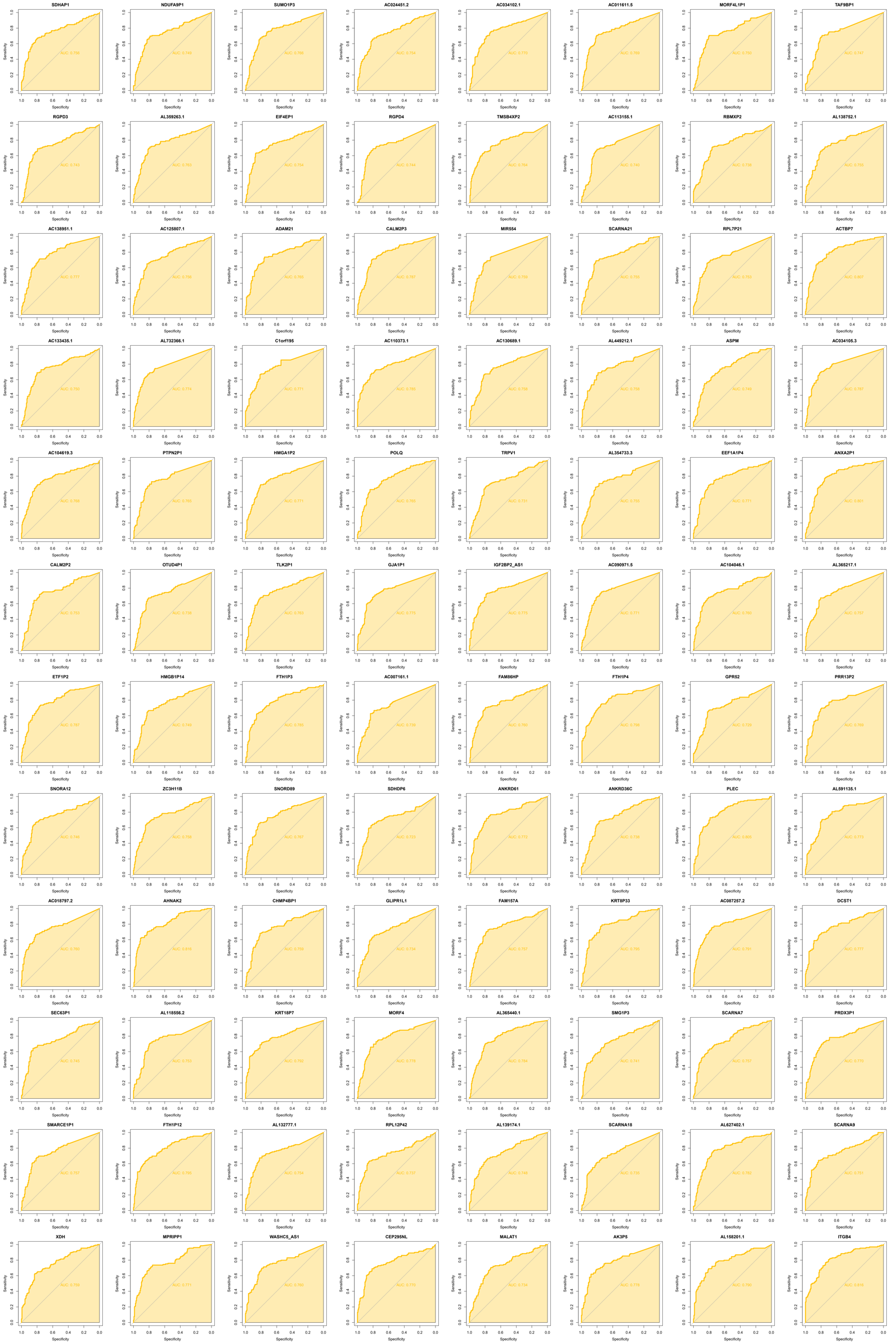

Supplement: Supplementary file 9 — Additional file 9: Fig. S9. ROC curves to predict hot-low and cold-high tumor conditions with DPIRGs [file 12967_2024_5590_MOESM9_ESM.pdf]

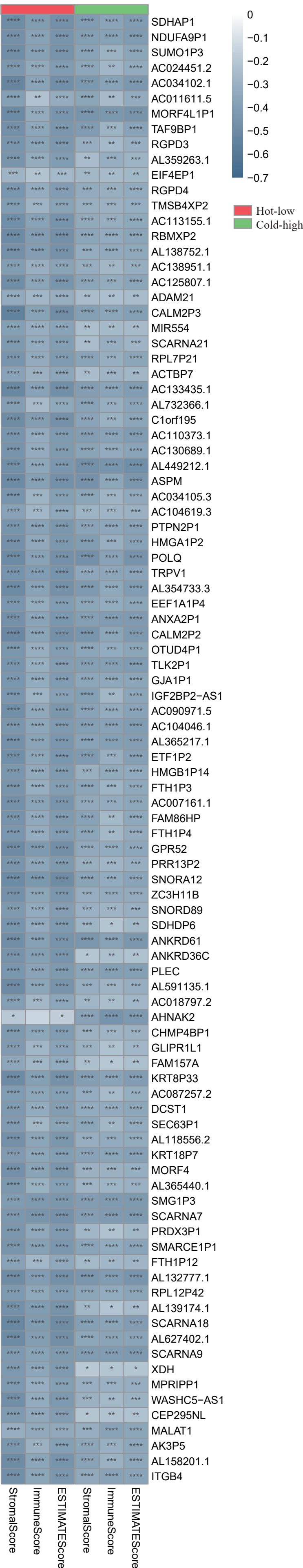

StromalScore

ImmuneScore

ESTIMATEScore

StromalScore

ImmuneScore

ESTIMATEScore

Supplement: Supplementary file 10 — Additional file 10: Fig. S10. Correlations between DPIRGs and three immune scores in the hot-low and cold-high groups [file 12967_2024_5590_MOESM10_ESM.pdf]

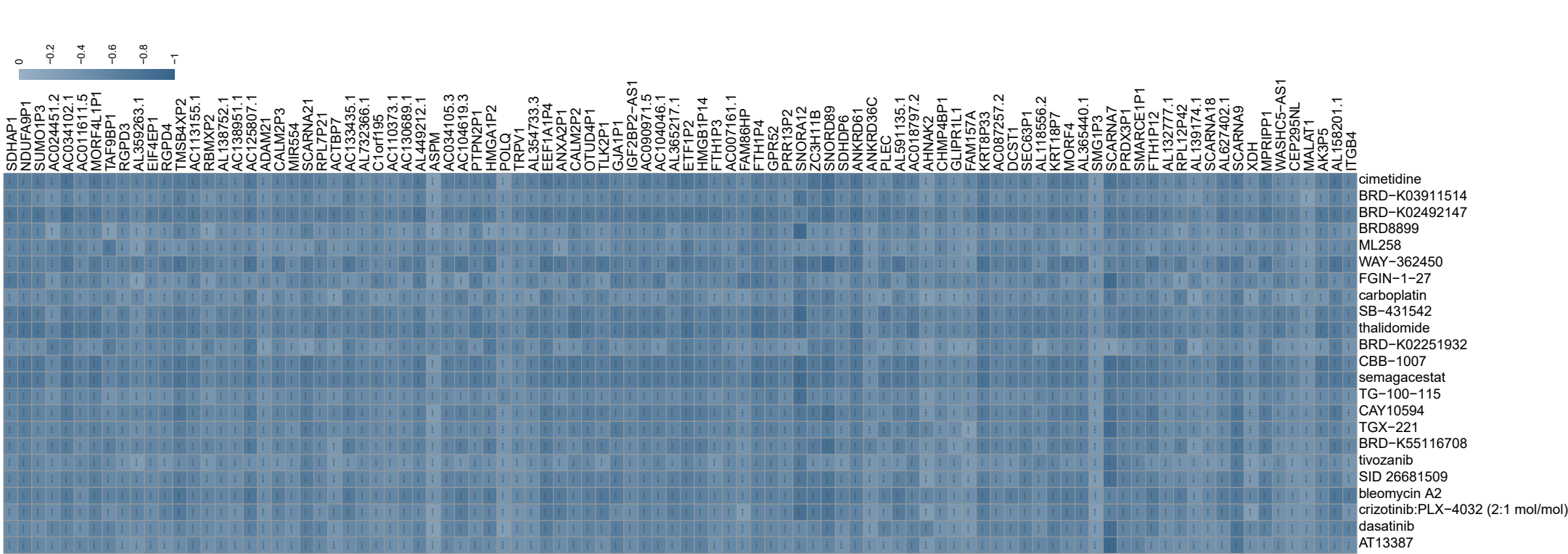

Supplement: Supplementary file 11 — Additional file 11: Fig. S11. The correlations between RNA expression levels of DPIRGs and predicted drug responses [file 12967_2024_5590_MOESM11_ESM.pdf]
