# Supplementary material for: Machine learning-based identification of biomarkers and drugs in immunologically cold and hot pancreatic adenocarcinomas
Source: J Transl Med. 2024 Aug 16;22:775. doi: 10.1186/s12967-024-05590-0 (PMC11328457; doi:10.1186/s12967-024-05590-0)
Supplement: Supplementary file 5 — Additional file 5: Fig. S5. Results of five machine learning methods based on mixed PAAD patients. A RF. B, C Boruta. D ANN. E SVM. F XGboost. [file 12967_2024_5590_MOESM5_ESM.pdf]

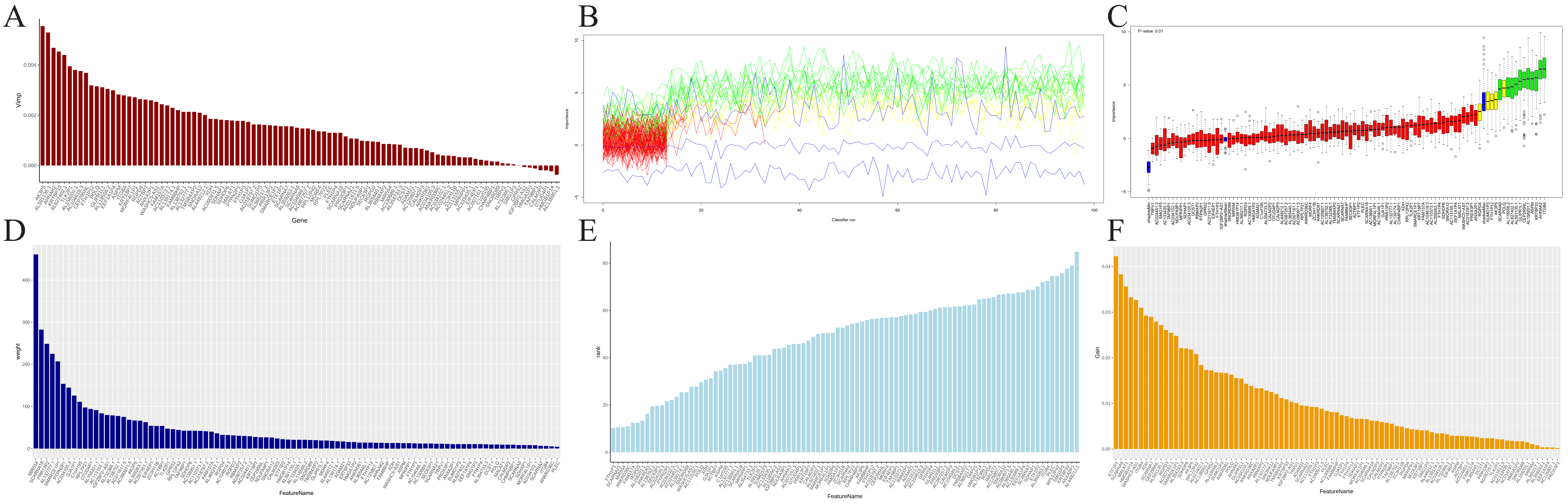
A
Vimp
Gene
B
Importance
Classifier run
C
P-value: 0.01
Importance
D
weight
FeatureName
E
rank
FeatureName
F
Gain
FeatureName
